# Water Sorption on Isoreticular CPO-27-Type MOFs: From Discrete Sorption Sites to Water-Bridge-Mediated Pore Condensation

**DOI:** 10.3390/nano14221791

**Published:** 2024-11-07

**Authors:** Marvin Kloß, Lara Schäfers, Zhenyu Zhao, Christian Weinberger, Hans Egold, Michael Tiemann

**Affiliations:** Department of Chemistry, Paderborn University, 33098 Paderborn, Germany

**Keywords:** metal–organic frameworks, MOF-74, IRMOF-74, open metal sites, water vapor sorption, hydrophobicity

## Abstract

Pore engineering is commonly used to alter the properties of metal–organic frameworks. This is achieved by incorporating different linker molecules (*L*) into the structure, generating isoreticular frameworks. CPO-27, also named MOF-74, is a prototypical material for this approach, offering the potential to modify the size of its one-dimensional pore channels and the hydrophobicity of pore walls using various linker ligands during synthesis. Thermal activation of these materials yields accessible open metal sites (i.e., under-coordinated metal centers) at the pore walls, thus acting as strong primary binding sites for guest molecules, including water. We study the effect of the pore size and linker hydrophobicity within a series of Ni^2+^-based isoreticular frameworks (i.e., Ni_2_*L*, *L* = dhtp, dhip, dondc, bpp, bpm, tpp), analyzing their water sorption behavior and the water interactions in the confined pore space. For this purpose, we apply water vapor sorption analysis and Fourier transform infrared spectroscopy. In addition, defect degrees of all compounds are determined by thermogravimetric analysis and solution ^1^H nuclear magnetic resonance spectroscopy. We find that larger defect degrees affect the preferential sorption sites in Ni_2_dhtp, while no such indication is found for the other materials in our study. Instead, strong evidence is found for the formation of water bridges/chains between coordinating water molecules, as previously observed for hydrophobic porous carbons and mesoporous silica. This suggests similar sorption energies for additional water molecules in materials with larger pore sizes after saturation of the primary binding sites, resulting in more bulk-like water arrangements. Consequently, the sorption mechanism is driven by classical pore condensation through H-bonding anchor sites instead of sorption at discrete sites.

## 1. Introduction

The concept of reticular synthesis [1] has proven to be one of the most fundamental concepts within the field of metal–organic frameworks (MOFs), a class of porous inorganic–organic hybrid materials. On the one hand, it provides a systematic approach to reduce the complexity of numerous MOFs [2,3] by simplifying their extended structures, reducing them to simple geometric building blocks, so-called secondary building units (SBUs). On the other hand, it guides chemists to rationally design new frameworks adopting the same framework connectivity, i.e., network topology, referring to the structure and connectivity of the underlying periodic network [4,5]. Substitution of geometrically identical units allows creation of precisely tailored pore environments by inclusion of new functionalities [6,7,8,9,10], pore size modification [6,10,11,12,13] or the use of different metal cations [14].

One of the most extensively studied examples of the applicability of this concept is CPO-27-*M* [15] (i.e., *M*_2_dhtp, dhtp = 2,5-dihydroxyterephthalate, also known as dobdc), also well-known as MOF-74-*M* [16]. Substitution of the dhtp-linker molecule [11,17,18,19] generates three-dimensional, isoreticular MOFs (i.e., IRMOF-74) with a honeycomb-like cross section and one-dimensional, cylindrical pores. This pore engineering concept resulted in multiple compounds with different pore sizes and functionalities such as *M*_2_dondc [20] (dondc = 1,5-dihydroxynaphthalene-2,6-dicarboxylate), *M*_2_bpp [11,17,18,19,21] (bpp = 3,3′-dihydroxy-[1,1′-biphenyl]-4,4′-dicarboxylate, also known as dobpdc) and *M*_2_tpp [11,17,19,21] (tpp = 3,3″-dihydroxy-2′,5′-dimethyl-[1,1′:4′,1″-terphenyl]-4,4″-dicarboxylate). Further studies showed that modification of the linker substitution pattern (i.e., from para- to meta-arrangement of carboxylic acid groups) results in frameworks with bended, ‘banana-like’ pore walls as in the cases of *M*_2_dhip [22] (dhip = 4,6-dihydroxyisophthalate, also known as *m*-dobdc) and *M*_2_bpm [21,22,23,24] (bpm = 4,4′-Dihydroxy [1,1′-biphenyl]-3,3′-dicarboxylate). All IRMOF-74 frameworks have accessible open metal sites [25] (i.e., under-coordinated metal centers) created by removing coordinating solvent molecules. These sites are exposed to the pores, acting as attractive sorption sites for various guest molecules such as CO_2_ [26,27,28,29] or water [28,30,31,32]. Therefore, these isostructural frameworks offer unique possibilities for sorption-based applications.

In order to evaluate the material performance, a deep understanding of the water sorption properties is needed, since applications either heavily rely on the absence (e.g., battery technology [33] and carbon capture [34,35]) or presence of water (e.g., proton conduction [36,37,38,39,40] and water harvesting [12,41]). Hence, a comprehensive knowledge of the interactions of water molecules inside the pores of the host framework is necessary. One powerful method to study the adsorption/desorption behavior of MOFs is water vapor sorption analysis. The isothermal water uptake of the sample is measured, typically applying either gravimetric methods (microbalance) [42,43] or manometric techniques [44,45]. For the latter method, we recently demonstrated the potential provided by high accuracy in the low-pressure range, i.e., for small amounts of water, studying the sorption mechanism of *M*_2_dhtp (*M*^2+^ = Co, Cu, Mg, Mn, Ni and Zn) [46,47], unveiling distinct differences of the sorption mechanisms, depending on the respective type of metal center.

In general, different sorption sites and unique sorption properties in MOFs [48,49] are a consequence of the chemical nature of the surface of these porous frameworks, gathered by a complex interplay of the hydrophilic inorganic building units and the (mostly) hydrophobic organic domains (i.e., linker molecules). However, discrete information on the polarity of the framework surface is obtained by determination of the inflection point α, i.e., the point at which half of the maximum adsorption is observed, of water adsorption steps. For polar materials, this point is shifted toward lower relative pressures (*p*/*p*_0_), when compared to less polar compounds [1,50]. Many MOF materials tend to lack sufficient hydrolytic stability [51,52], which is an immediate consequence of the dynamic nature of the coordinative bonds in competition with water molecules [53]. Nevertheless, the above-discussed isoreticular approaches to modulate the pore properties in MOFs make them interesting materials for detailed water sorption studies. Qian et al. [10] systematically studied UiO-66(Zr) by independently analyzing the effect of the pore wall polarity (via linker functionalization) and pore size (via incorporation of larger linker moieties) on its water sorption behavior. Linkers with higher hydrophilicity revealed a pronounced shift of the observed sorption step toward lower relative pressures, suggesting stronger water–framework interactions. Similar observations were also made for other MOFs [13]. However, incorporation of highly polar linkers drastically altered the water sorption behavior, changing the shape of the observed water sorption isotherm (from type IV to type I [54]), suggesting highly attractive conditions for water adsorption, as similarly observed for porous carbon [55]. In contrast, hydrophobic groups only led to severely reduced water uptake without further visible changes. Further, pore size-dependent shifts of the sorption step toward higher pressures were observed, indicating that micropore filling is highly affected by the given pore diameter and the increased size of hydrophobic domains, since pore condensation relies on water cluster formation, as similarly observed for other MOFs [56,57,58].

We present a comprehensive study on the effects of pore size and linker substitution pattern on the water sorption mechanism and water arrangement within the one-dimensional, cylindrical pores of a series of isoreticular MOFs (i.e., Ni_2_*L*; *L* = dhtp, dhip, dondc, bpp, bpm and tpp). We utilize thermogravimetric analysis (TGA) and Fourier transform infrared (FTIR) spectroscopy to monitor the interactions of water with its environment in the confined space. In addition, we apply solution ^1^H nuclear magnetic resonance (NMR) spectroscopy to monitor the number of defects from missing linker molecules. Finally, manometric water vapor sorption analysis is used to study the adsorption/desorption behavior of the IRMOF-74 series.

## 2. Materials and Methods

### 2.1. Linker and MOF Synthesis

All *M*_2_*L* MOF materials (i.e., Ni_2_dhtp [59], Ni_2_dhip [22], Ni_2_dondc [20], Ni_2_bpp [11], Ni_2_bpm [11], Ni_2_tpp [11]) were synthesized under solvothermal conditions according to modified reported procedures (details are given in the Supplementary Materials). Solvent was removed by heating under dynamic vacuum conditions to obtain desolvated samples, which were stored in a glove box under Ar atmosphere until further use. All products were characterized by powder X-ray diffraction (XRD) and compared with literature results, ensuring structural integrity with no evidence of crystalline impurities.

H_4_dhtp [46,60], H_4_dondc [20], H_4_bpp [17,61] and H_4_tpp [17,61] linker molecules were synthesized according to modified literature procedures (details are given in the Supplementary Materials). All other chemicals (including H_4_dhip and H_4_bpm) and solvents were purchased from commercial suppliers and used without further purification. All air- or water-sensitive reactions were carried out under standard Schlenk techniques using a dry argon atmosphere.

### 2.2. Preparation of Hydrated Samples

The desolvated samples were placed in a closed vessel with a separate water reservoir (1 mL per sample, ca. 10 to 15 mg) to load them via the gas phase at 40 °C for 24 h. Afterwards, we refer to them as hydrated samples (Ni_2_*L*-hyd).

### 2.3. General Characterization Techniques

Powder X-ray diffraction (XRD) data were collected on a Bruker D8 Advance diffractometer (Bruker, Karlsruhe, Germany) with a step size of 0.02° and a counting time of 3 s per step. Patterns are normalized (data range from 0, 1) to the most intense reflection for better comparison of relative intensities. ^1^H and ^13^C nuclear magnetic resonance (NMR) spectra were recorded using Bruker Advance 500 and Bruker Ascent 700 spectrometers (Bruker, Ettlingen, Germany). Chemical shifts were calibrated to the resonance of residual non-deuterated solvent. For defect analysis, small amounts of the samples (ca. 3 to 5 mg) were diluted overnight in 600 µL of a solvent mixture prepared from dmso-d_6_ (10 mL) and deuterium chloride (20 wt% in D_2_O; 0.5 mL). Thermogravimetric analysis (TGA) was performed using a TGA/DSC1 STAR System thermobalance from Mettler-Toledo (Gießen, Germany). Samples were placed in a 70 µL corundum crucible. Measurements were performed under a constant nitrogen gas flow (50 mL min^−1^, purity 5.0) in a temperature range from 40 °C to 800 °C using a heating rate of 10 °C min^−1^.

### 2.4. Fourier Transform Infrared (FTIR) Spectroscopy

FTIR spectroscopy was performed using a Bruker Vertex 70 spectrometer (Bruker, Ettlingen, Germany) in annual total reflection (ATR) mode utilizing the Platinum ATR unit A225 with a diamond ATR crystal. Powdered samples were pressed on the crystal during the measurement. After the measurement of hydrated samples, an automatic baseline correction was applied using the OPUS 7.2.1 software package. The correction was performed using a concave rubberband correction, with 10 iterations and 32 baseline points. Gaussian least square fits of the water stretching band were performed individually using the peak analyzer function of the Origin23b software package. First, the data within the range from 4500 to 2400 cm^−1^ were normalized (data range from 0.1) to the maximum absorbance. For the fitting procedure, the data from 3900 to 2400 cm^−1^ were selected. Baseline correction was performed, using a linear baseline. Then, three peaks were set with the starting positions near 3560 cm^−1^ (multimer water), 3380 cm^−1^ (intermediate water) and 3210 cm^−1^ (network water). Only positive peak areas were allowed, while the previously corrected baseline was set constant. No further restrictions were used. It was noted that the fit results possess a small error, caused by the overlap of the aromatic C-H vibration of the linker molecules with the water stretching bands.

### 2.5. Sorption Analysis

#### 2.5.1. Nitrogen Physisorption Measurements

N_2_ physisorption analysis was performed with a Quantachrome Autosorb 6B (Quantechrome Instruments, Boca Raton, FL, USA) at 77 K. The relative pressure range suitable for the BET area calculation was determined using the Rouquerol [62] criteria. Total pore volumes were determined from the uptake at *p*/*p*_0_ ≈ 0.9. Activation of the samples was performed in a stepwise manner. The desolvated samples were transferred to the measurement cell and dispersed in methanol twice for 45 min. After each step, the solvent was removed under dynamic vacuum. The pre-dried samples were degassed for approximately 17 h applying the following procedure: Samples were heated from room temperature to 60 °C with a heating rate of 2 °C min^−1^ after which the temperature was held for two hours. Then, the sample was heated to 100 °C with the same heating rate (2 °C min^−1^) and the temperature was held for another two hours. Finally, the sample was heated (2 °C min^−1^) to 150 °C and the temperature held for an additional 12 h, after which the sample was allowed to cool to room temperature. Pore size analysis was accomplished with the supplied AS-Multistation 2.01 software package using the NLDFT data-based method (N_2_ at 77 K on silica, cylind. pore, NLDFT adsorption branch data kernel), treating the samples as silica.

#### 2.5.2. Water Vapor Sorption Measurements

Water vapor sorption experiments were performed on a 3Flex instrument (Micromeritics, Unterschleißheim, Germany) at 298 K (25 °C) with double distilled (and degassed) water. Activation of the samples was performed in a stepwise manner, applying the same procedure as mentioned in the N_2_ physisorption section.

## 3. Results and Discussion

### 3.1. General Characterization

**Figure 1:**
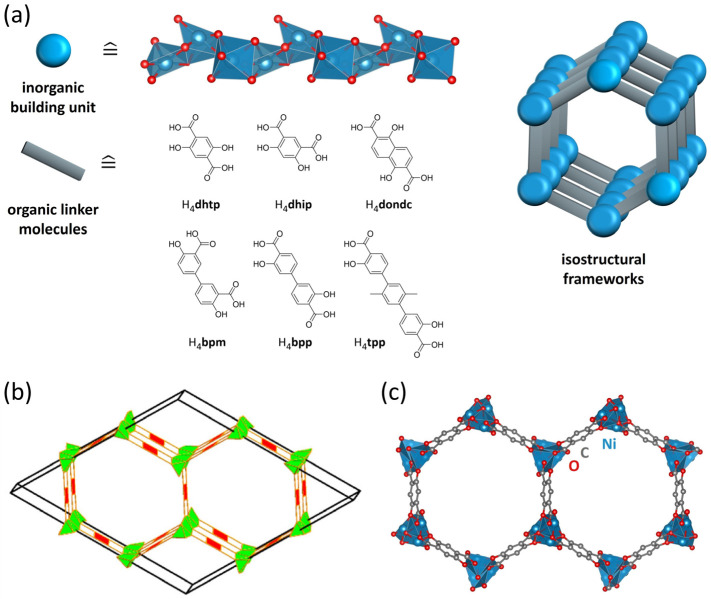
(**a**) Illustration of the inorganic building units and used linker molecules in this research, forming isostructural Ni_2_*L* frameworks. (**b**) Network topology of Ni_2_dhtp (msf, reprinted from ref. [3]) and (**c**) representation of the honeycomb-like cross section along the crystallographic *c*-axis, showing Ni_2_dhtp as an example.

All members of our nickel-based frameworks, i.e., Ni*_2_L* (*L* = linker molecule), were prepared using six linker molecules, each presenting either a different substitution pattern (para- vs. meta-position of carboxylate groups) or different sizes, resulting in isoreticular materials with tunable size and pore wall polarities (Figure 1).

**Figure 2:**
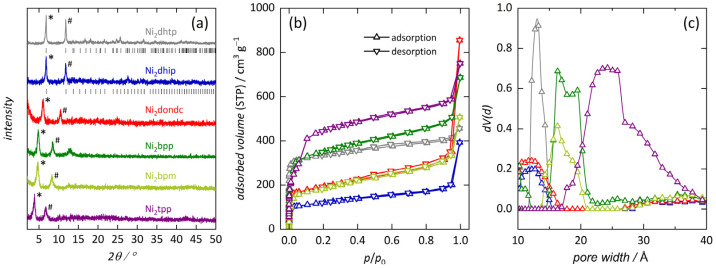
XRD patterns of Ni_2_*L* frameworks (**a**). The most intense reflections represent the (110) and (300) lattice planes (marked with * and # respectively), being shifted toward lower angles with increasing pore diameter. Patterns are normalized and compared to literature data where accessible [22,63]. N_2_ physisorption isotherms of Ni_2_*L* (**b**) measured at 77 K and their respective NLDFT-based pore size plots (**c**).

Powder X-ray diffraction (XRD) data of the prepared frameworks (i.e., Ni_2_*L*) are in agreement with literature data [11,17,20,21], confirming the periodic, three-dimensional framework structure with a honeycomb-like cross section, with no evidence of crystalline impurities in any of the prepared samples (Figure 2a). The respective (110) lattice plane distances, characteristic for the perpendicular arrangement of the one-dimensional cylindrical pores, indicate the expected pore diameters in the range 13 … 23 Å (Table 1). We note that the broad reflections for most materials indicate small crystallite size, while the signal-to-noise ratio is attributed to sample preparation and the crystallinity of the frameworks (longer measurement times were avoided to minimize the influence of humidity).

N_2_ physisorption analysis enabled us to confirm the permanent porosity of all products, revealing type I sorption isotherms [64] for most frameworks in our series, consistent with the existence of micropores (Figure 2b). In contrast, Ni_2_tpp reveals a type IV(b) isotherm shape typical of mesoporous materials with pore diameters below 40 Å. The suitable pressure range for BET area determination was selected using the Rouquerol [62] criteria. The high specific surface areas (560 … 2320 m^2^ g^−1^) and large specific pore volumes (0.38 … 1.03 cm^3^ g^−1^, at *p*/*p*_0_ ≈ 0.9) of the networks mostly align with literature data (reference data only for Mn_2_dondc) [17,20,21,22,23]. We note that the comparable low surface areas for some materials hint toward the existence of amorphous byproduct, while the increase in the adsorbed volume at high relative pressures generally indicates interparticle porosity as a consequence of small crystallite size Observed differences for Ni_2_dhip and Ni_2_bpm are slightly larger. Pore sizes analysis (Figure 2c) is in good agreement with previous studies [21] and our XRD results, verifying that the pore size within our series approximately doubles from Ni_2_dhip to Ni_2_tpp (see Table 1, details are shown in the Supplementary Materials; NLDFT-based fits displayed in Figures S6–S11). However, the uniformity of the pore sizes is seemingly affected by the average pore diameters, resulting in a broadening of the distribution curve, in agreement with the expectation that larger inorganic moieties negatively influence the frameworks crystallinity.

Recent studies analyzed formate defects in Ni_2_dhtp stemming from decomposed DMF (DMF = *N*,*N*-dimethylformamide) solvent molecules. They found that the number of defect sites increases with the metal-to-linker ratio [65]. Therefore, we applied ^1^H NMR spectroscopy to analyze potential defects within our series. Samples were diluted in a solvent mixture of dmso-d_6_ and deuterium chloride (20 wt% in D_2_O). We note that acid-caused decomposition of formate anions [66] is a possible error source. However, we suggest that the impact of this is comparably low due to the reasonably low acid concentration used for decomposition. NMR spectra of diluted frameworks are shown in the Supplementary Materials (Figures S12–S17). Respective ratios of the linker molecules and formate anions (For^−^), or acetate (OAc^−^) anions for Ni_2_dondc, were calculated considering the different charges of the coordinating ligands, i.e., one linker (*L*^4−^) must be replaced by four For^−^/OAc^−^ ions (capping ligand) to maintain charge balance. This enabled us to determine the respective sum formula for our materials (see Table 2). We found that even for low metal-to-linker ratios (e.g., Ni_2_dhtp) the amount of incorporated formate ions is significant (i.e., 0.62, suggesting 0.16 dhtp^4−^ molecules are replaced). However, we see the reported trend of increased defect sites with increasing metal-to-linker ratios for all materials in our series. The precise amount of the capping ligands depends on the nature of the linker molecule (acidity, solubility, …) and the precise reaction conditions. Ni_2_dhip revealed the largest differences between individually synthesized samples, indicating that its synthesis is more sensitive to minor changes compared to the other materials in this study. Further, we found formate ions in diluted samples of Ni_2_dondc (Figure S18), which we attribute to thermally decomposed DMF used for the replacement of *N*-methyl-2-pyrrolidone (NMP) solvent molecules. We suggest that these post-synthetically generated formate ions do not affect the defect degree of the pristine framework but coordinate to the open metal sites. Hence, these molecules can be replaced by additional solvent exchange procedures, as performed prior to sorption analysis. However, this suggests that the determined formate ion concentration in diluted samples is slightly overestimated as a consequence of residual DMF solvent molecules. Nevertheless, the amount of incorporated capping ligands clearly is another contributing factor for the broadening of the pore size distribution (see above), in agreement with the negative effect of defect sites on the materials’ crystallinity. For the sake of simplicity, all materials are referred to Ni_2_*L*, and presented defects are discussed when necessary.

### 3.2. Thermal Analysis

**Figure 3:**
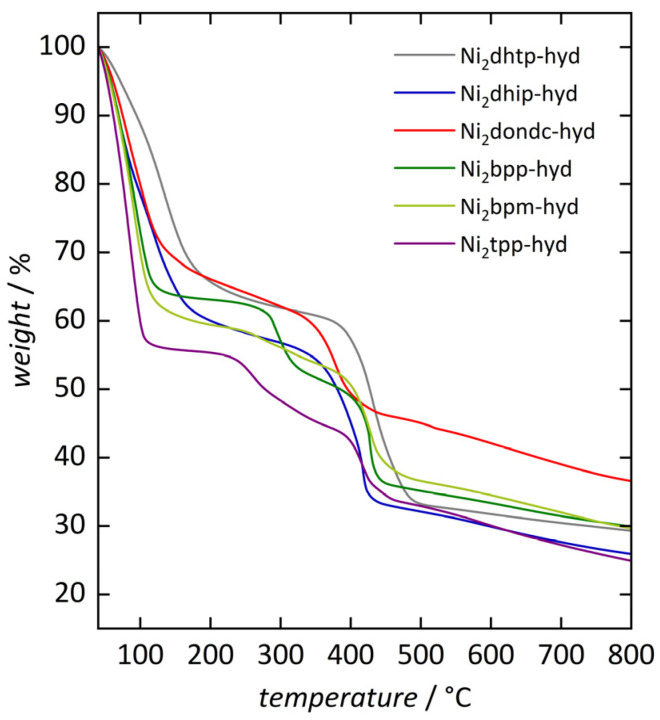
Thermogravimetric analysis of the hydrated materials (Ni_2_*L*-hyd) from 40 to 800 °C under N_2_ atmosphere (heating rate: 10 °C min^−1^).

To evaluate the thermal stability of our frameworks and provide an ideal starting point for our hydration/dehydration experiments, we performed thermogravimetric analysis (TGA) of the hydrated frameworks (Ni_2_*L*-hyd, Figure 3). Samples were prepared by loading the desolvated materials with water through the gas phase. Therefore, samples were placed in a closed vessel with a separate water reservoir to load them with water above room temperature (i.e., 40 °C) for 24 h; for more details, see Section 2.2.

In general, framework decomposition is observed in the range 350 … 450 °C, comparable with previous reports [17,21]. However, the above-mentioned formate/acetate defects affect the thermal stability of the frameworks. Prior to framework decomposition, an additional smaller mass loss step around 250 °C (Ni_2_tpp) and 300 °C (Ni_2_bpp) is observed for some frameworks. This is in accordance with previous studies on defect engineering in Ni_2_dhtp, revealing additional decomposition steps, becoming more pronounced with higher defect degrees [67]. Our results on Ni_2_bpp and Ni_2_tpp are comparable to previous studies, although the here-observed steps are more pronounced and, for Ni_2_tpp, shifted toward lower temperatures [17,21]. These differences are likely to arise from slightly different synthetic approaches (here: mostly DMF [59] vs. DMF/EtOH/water (1/1/1) mixture with lower Ni-to-*L* ratios), resulting in varying defect sites [65]. TGA data further indicate that the mass stability, i.e., formation of a stable plateau, depends on the defect degree, as increasing amounts of For^−^/OAc^−^ lead to the loss of a stable mass plateau after the water loss step and finally, an additional pronounced decomposition step. We note that our results for Ni_2_dhtp deviate from our previous studies [46], where larger amounts of OAc^−^ ions were incorporated (stemming from the metal source), visible in an additional decomposition step prior to the collapse of the framework. This suggests that the herein-found formate defects do not significantly alter the thermal stability of Ni_2_dhtp, agreeing with the above-referenced studies.

Next, we determined the amount of adsorbed water in all hydrated Ni_2_*L*-hyd materials by considering the first mass loss step between 40 and 250 °C. (We note that the adsorbed amount of water is generally underestimated due to its strong bonding to the Ni^2+^ sites, being slowly desorbed over a broad temperature range as well as during framework collapse [32,46]). All materials reveal similar values (21 … 25% weight loss, Supplementary Materials Table S1), slightly increasing with pore size. However, Ni_2_tpp deviates from this trend, indicating that some loosely bonded water molecules could desorb during sample preparation, as indicated by a less stable sample mass during measurement setup. In general, the dehydration step shifts toward lower temperatures with increasing pore diameter, indicating water–framework interactions become less attractive and the adsorbed water becomes more comparable to bulk water.

### 3.3. FTIR Spectra of Hydrated Samples

Our results presented above indicate the expected effect of the channel size on water–framework interactions and hint toward different water arrangements in the one-dimensional channels. Thus far, infrared spectroscopy is frequently used to analyze the interactions of water molecules at surfaces (e.g., inverse micelles [68,69]) or within pores (e.g., mesoporous silica [45,70] and MOFs [46,71]). This possibility arises from the deconvolution of the O–H stretching vibration, a superposition of vibrational bands, typically located in the range of 2800 to 3700 cm^−1^ [72]. Least squares fitting of three Gaussian profiles enables us to determine the respective contributions (peak areas) of the different vibrational bands assigned to water in a particular H-bonding environment [45,68,69]. Brubach et al. [72] designated these water molecules as different types with respect to their coordination numbers (CN), i.e., numbers of H-bonding partners. These three types are termed ‘network water’ (NW), ‘intermediate water’ (IW) and ‘multimer water’ (MW), as their respective stretching frequencies reveal a red shift, i.e., toward lower wavenumbers, with an increasing number of intermolecular interactions. NW resembles highly H-bonded water molecules interacting strongly with their respective environment, forming roughly four H-bonds (CN ≥ 4) with adjacent atoms. These structures are often referred to as ‘ice-like’ arrangements, possessing oscillation frequencies around 3260 cm^−1^. As the number of interactions decreases, IW water populations form, showing frequencies around 3460 cm^−1^. These populations offer a ‘bulk-like’ water arrangement of more dynamic water molecules. Lastly, MW resembles poorly connected water molecules, often found at interfaces, forming only few H-bonds (as found in dimeric/trimeric structures). These MW water molecules are also referred to ‘dangling’ water molecules, being a consequence of their isolation, i.e., low connectivity, to other water molecules. MW typically shows vibration frequencies located around 3600 cm^−1^.

**Figure 4:**
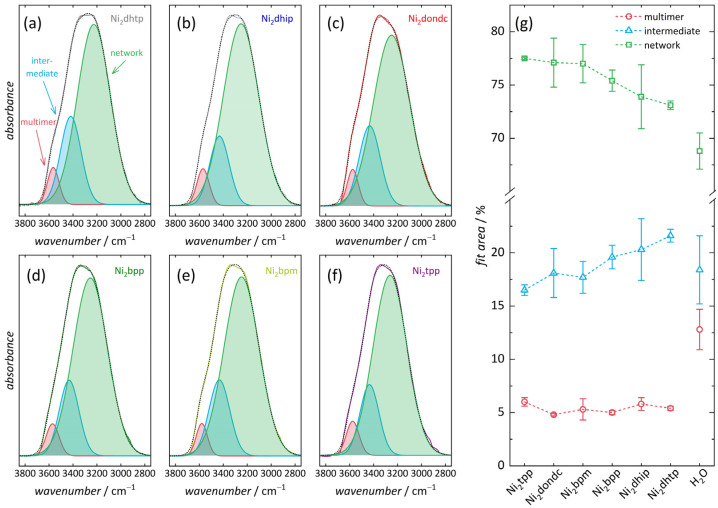
Deconvolution of the FTIR O–H stretching vibration band of water in hydrated Ni_2_L-hyd samples (**a**–**f**) by least squares fitting (Dotted lines: sums of the three Gaussians). Relative contributions of multimer, intermediate and network water in comparison with a pure liquid water film (**g**).

We carried out Fourier transform infrared (FTIR) spectroscopic measurements in attenuated total reflection (ATR) mode on hydrated samples (i.e., Ni_2_*L*-hyd) to analyze the effect of the pore size and linker substitution pattern on the arrangement and interactions of adsorbed water molecules. The spectra reveal the integrity of the organic linker molecules as well as the presence of three of the characteristic vibration bands of water, i.e., the stretching band (3750 … 2800 cm^−1^), the bending band (1700 … 1650 cm^−1^), and the libration band (1000 … 400 cm^−1^) (see Supplementary Materials Figures S19–S24) [72]. Next, least squares fitting was performed for a pure liquid water film located at the surface of the ATR crystal (Supplementary Materials Figure S25), as a reference to quantify the relative contributions of the different water vibrations. We observed a contribution of 13% from multimer water (3535 cm^−1^), stemming from water in the liquid film that is located at either of the two interfaces (ATR diamond crystal or air) [46]. Further, contributions of 18% from intermediate water (3405 cm^−1^) and 69% from network water (3251 cm^−1^) indicate that most of the water molecules not located at the interfaces form multiple H-bonds with surrounding molecules. All Ni_2_*L*-hyd samples possess significantly lower contributions for the multimer peak (5–6%) (Figure 4 and Supplementary Materials Table S2) when compared to the pure water film. This suggests that, similar to *M*_2_dhtp [46], water molecules in these confined environments show a higher tendency for H-bonding than interface-near water molecules of a pure liquid water film. This further suggests that additional water molecules interact strongly with the coordinating water molecules, located at the primary binding sites, and with oxygen atoms of the inorganic building unit, giving rise to further H-bonding possibilities. Further, all Ni_2_*L*-hyd networks show slightly higher contributions from network water (73–78%) as well as similar contributions from intermediate water (17–22%) when compared to the pure water film, suggesting that multiple water molecules form a highly connected H-bonding network.

We note that the observed relative contributions of the three vibrational bands in Ni_2_dhtp-hyd differ from earlier results [46]. As mentioned above, this is explained by different synthetic procedures, resulting in more defect sites caused by significant incorporation of acetate anions (from the metal source). These capping ligands alter the pore surface, resulting in a less uniform pore wall structure, causing less attractive interactions of water with the framework because of reduced H-bonding possibilities. Further, the precise adsorption mechanism is affected (as discussed below). As stated above, the Ni_2_dhtp formed here possess fewer defects, which is typical for reactions applying small metal to linker rations [65]. Consequently, a more uniform pore wall structure and stronger interactions with more H-bonding opportunities are found. Expecting crystallographically defined water positions in Ni_2_dondc, similarly for Mn_2_dondc [20], we found different MW (lowest observed) and NW (second highest) contributions, suggesting a similar number of water molecules within the hydrated framework (ca. five molecules per Ni^2+^). However, defects within the other frameworks seem to play a minor role for these interactions, since most water molecules form multiple H-bonds even in the presence of significant amounts of capping ligands. This indicates that potential binding sites near the pore walls are significantly less attractive for frameworks with larger hydrophobic domains. We hypothesize that the high NW contributions found therein stem from inner pore, more bulk-like water, rather than from interfacial water (i.e., MOF–water interactions as found for *M*_2_dhtp [46]).

Consideration of the respective wavenumbers $\tilde{v}$ (peak positions, see Supplementary Materials Table S3) of the three Gaussian contributions allows us to gain more information on the O–H bond situation of confined water. When compared to the pure water film, all Ni_2_*L*-hyd samples show a blue shift (i.e., toward higher $\tilde{v}$) of the multimer and intermediate water peak. Observed shifts are comparable for all materials except for Ni_2_dhtp-hyd, which reveals a less pronounced blue shift, generally indicating O–H bond strengthening upon hydration. In addition, most compounds reveal no significant changes of the network water peak. However, Ni_2_dhtp-hyd reveals a red shift (toward lower $\tilde{v}$), indicating weakening of the O–H bonds in NW water stemming from highly confined water molecules. For Ni_2_tpp-hyd, the opposite, i.e., blue shift, is found, being a consequence of less confined water molecules.

The local H-bonding situation inside the one-dimensional pores of hydrated frameworks is another aspect to bear in mind to monitor pore-size-dependent differences. Information on this is derived from the respective differences between the peak center positions *x* and *y* of different vibrational bands, i.e., from the wavenumber splitting Δ$\tilde{v}$_xy_ (Table 3). Smaller Δ$\tilde{v}$ values suggest a higher symmetry in the local H-bonding of water, indicating more mobile water molecules with high rotational degrees of freedom and vice versa [73,74]. We determined higher values for all Ni_2_*L*-hyd materials than found for a pure water film. This indicates a higher degree of H-bonding asymmetry, which is consistent with the expectation of confined water molecules. Despite the similar trend, the precise Δ$\tilde{v}$ values differ for any given compound. For Ni_2_*L*-hyd materials with smaller pore diameters, i.e., *L* = dhtp, dhip and dondc, generally higher wavenumber splitting is found. In the particular cases of Ni_2_dhtp [32] and Ni_2_dondc [20], this is in accordance with the expectation of crystallographically defined water positions in a fixed, highly asymmetric local H-bonding situation. However, frameworks with larger pore diameters, i.e., *L* = bpp, bpm and tpp, show the opposite trend, revealing lower Δ$\tilde{v}$ values. H-bonding becomes more symmetric, and thus are more comparable with bulk water because of the less pronounced confinement effects. These observations are in accordance with our TGA experiments and previous results on mesoporous silica revealing similar effects of the pore size [70], highlighting a more bulk-like behavior with more flexible water molecules. Considering these results, the members with larger pores (Ni_2_*L*, *L* = bpp, bpm and tpp) should reveal a water sorption behavior that mostly resembles classical pore condensation (i.e., water cluster formation), rather than the occupation of discrete positions and confinement effects (as expected for the smaller pores).

### 3.4. Water Vapor Sorption

To prove our hypothesis regarding the effect of the pore size on the sorption mechanism, we analyzed the hydration/dehydration behavior of the Ni_2_*L* with manometric water vapor sorption measurements at 25 °C. We carried out two consecutive adsorption/desorption cycles without additional thermal activation in between the two cycles (i.e., samples were not removed from the device), since our previous water sorption studies on *M*_2_dhtp [46,47] highlighted that coordination of water to the open metal site during the first sorption isotherm results in different starting conditions ascribed to strongly bound (chemisorbed) water molecules. These cannot be removed during the desorption process without additional thermal activation.

We observed an overall higher total uptake during the second adsorption/desorption cycle when compared to the first cycle for Ni_2_dhtp. This is explained by a partial degradation of the frameworks resulting in the formation of additional open metal sites caused by cleavage of metal–linker bonds [42,46,47] during the time-consuming measurements (several days). Results on Ni_2_dhip indicate a poor hydrolytically stability since we observe a decrease in water uptake during the first desorption isotherm, reflected by the crossing of the adsorption and desorption branch and large equilibrium times, making it impossible to further evaluate its water sorption behavior in detail. On the other hand, Ni_2_*L* frameworks containing larger linker moieties (i.e., *L* = dondc, bpp, bpm and tpp), and thus possessing larger pores, reveal a severely decreased total water uptake during the second cycle, indicating that water molecules affect the framework’s longevity. All observations are consistent with the decreased crystallinity of all samples after water sorption experiments (Supplementary Materials Figure S26), while most materials maintain a larger degree of their initial pore ordering (except for Ni_2_dondc, which reveals complete loss of crystallinity). Since all *M*_2_*L* frameworks adsorb water at low relative pressure (*p*/*p*_0_ < 0.1) due to highly attractive interactions with vacant coordination sites, we displayed all isotherms, both in a linear and in a semi-logarithmic representation, to analyze differences in the sorption behavior. In addition, first derivatives of the isotherms (δ*V*/δ(*p*/*p*_0_)) were calculated to highlight the distinct water sorption steps in all isotherms (see Supplementary Materials Figures S27–S32) (Missing data points below *p*/*p*_0_ = ca. 0.002, except in the first adsorption isotherms, are due to technical experimental restrictions).

**Figure 5:**
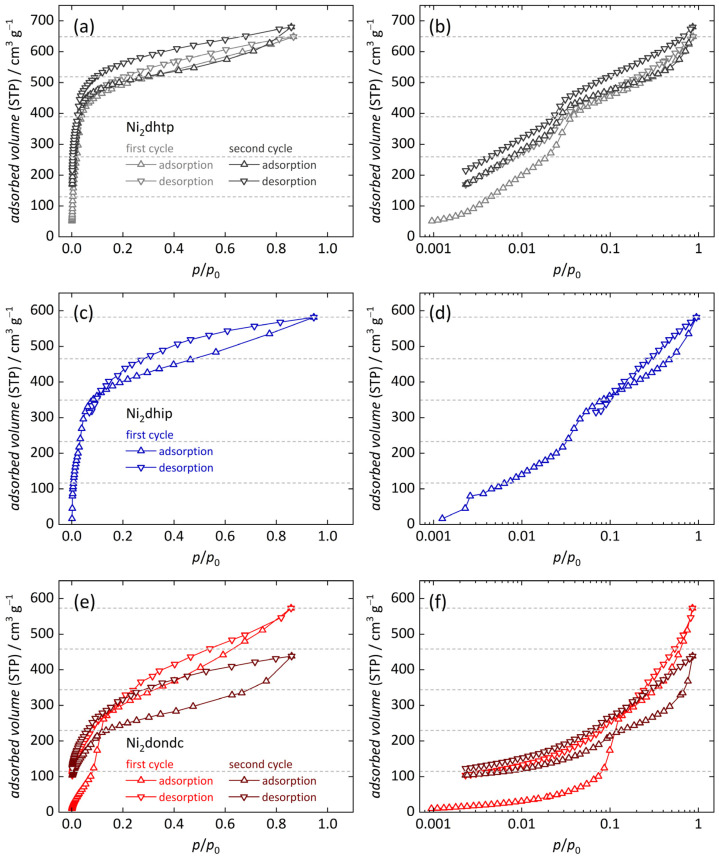
Water vapor sorption isotherms (25 °C) of Ni_2_dhtp, Ni_2_dhip and Ni_2_dondc (two consecutive adsorption/desorption cycles). Data are shown at linear scale (**a**,**c**,**e**) and in semi-logarithmic representation (**b**,**d**,**f**). Horizontal lines mark 20%, 40%, 60%, 80% and 100% of the total water uptake during the first cycle.

The water sorption isotherms of Ni_2_dhtp (Figure 5a,b), reveal similar characteristics as observed in previous studies on *M*_2_dhtp [46,47], revealing a type I [54] water sorption isotherm. However, differences arise as both materials are compared with each other. Accordingly, uptake of the first 20% (with respect to the total uptake) occurs at particularly low pressures (*p*/*p*_0_ < 0.003), attributed to the coordination of water to open meal sites, even though the desorption data are inconclusive with respect to the irreversibility of this process, i.e., missing low-pressure data due to technical limitations. The further course of the isotherms suggests a slightly different sorption mechanism: The observed sorption step at *p*/*p*_0_ = 0.03 … 0.05 in this study corresponds to the uptake of one rather than two water molecules in our previous study (ca. 20%, resulting in 60% of the total water uptake vs. 40%, resulting in 80%) and presumably results in the formation of a uniform water cluster along the pore walls of the framework, being in agreement with the water sorption mechanisms found for most other metals within the *M*_2_dhtp series (except *M* = Cu) [46,47]. As mentioned above, these findings result from varying defect degrees. Significant incorporation of acetate anions (and presumably capping ligands in general), results in less uniform pore walls within the Ni_2_dhtp framework, and thus less attractive interactions of water with the pore walls, i.e., via H-bonding with the phenylene backbone at the proposed third adsorption site. Consequently, the adsorption mechanism is altered, making the adsorption of two water molecules more attractive to create a metastable state (i.e., preliminary pore saturation). However, the further cause of our isotherm suggests that the here observed formation of a continuous water layer along the pore walls seems to be a meta-stable state, since gradual absorption of additional water molecules occurs afterwards until complete hydration is observed, without any sign of additional sorption steps. We attribute this to stronger Ni–O interactions in the inorganic SBU when compared to the other members of the *M*_2_dhtp series [47], affecting atomic charges on the metal center, thus preferring a square planer coordination environment of Ni^2+^. Consequently, chemisorption of water molecules to the metal center as well as H-bonding with framework O atoms becomes less attractive, i.e., larger H-bonding distances, causing similar adsorption energies of both sorption sites. Subsequent occupation of the third site (near the phenylene backbone) results in more water molecules arranged in the confined environment. Hence, preliminary pore saturation is observed at lower relative pressures when compared to *M*_2_dhtp [46,47]. Then, uptake of additional water molecules with increasing vapor pressure (i.e., during adsorption) is enabled by relaxation of previously adsorbed molecules, providing additional space while decreasing their respective mobility. This phenomenon creates a hysteresis because of an increased density of the adsorbed water, which is irreversible during the desorption, i.e., with decreasing pressure. We previously ascribed this effect to the alternating hydrophobic (phenylene) and hydrophilic (coordinated metal centers) domains within *M*_2_dhtp [46,47], which is in accordance with similar effects found for other porous materials possessing larger and/or more hydrophobic pores [45,75,76,77,78,79,80,81,82,83,84]. The total water uptake after the first adsorption branch was determined to be 0.51 ± 0.06 g g^−1^, which is in agreement with earlier studies [17,85]. Our findings on Ni_2_dhtp indicate that the precise sorption mechanism can be tailored by selective incorporation of defects, giving rise to tailor-made water sorption properties [59,65,67,86]. We will carry out further experiments in the future to validate this hypothesis and confirm if this is a general property of the *M*_2_dhtp framework series.

Continuing with Ni_2_dhip, the water sorption isotherms reveal a similar shape compared to Ni_2_dhtp (Figure 5c,d). Slight differences within the low-pressure range *p*/*p*_0_ < 0.05 suggest that the less uniform structure of the pore walls because of the different substitution patterns (i.e., para- vs. meta-arrangements of the carboxylate groups) has only a minor influence on the possible sorption sites within the one-dimensional pores. In addition, the total water uptake is slightly lower (0.41 ± 0.06 g g^−1^), which is probably due to the decreased void spaces, affecting especially sorption sites near the pore center. We note that these effects could also stem from the observed higher defect-degrees within Ni_2_dhip when compared to Ni_2_dhtp. As mentioned above, the framework decomposes during the first desorption branch, making it impossible to analyze any effects of the chemisorbed water on the second cycle.

The water sorption isotherms of Ni_2_dondc (Figure 5e,f) offer fewer similarities to the previously discussed Ni_2_dhtp, displaying a type IV [54] water sorption isotherm. Here, water adsorption proceeds less at low relative pressures (*p*/*p*_0_ < 0.1) and is attributed to the irreversible saturation of the open metal sites, i.e., chemisorption to the primary binding sites (ca. 20% of the total uptake remain adsorbed after completed desorption). Following this, a pronounced adsorption step at *p*/*p*_0_ = 0.1 is detected, accountable for a majority of the water uptake. This indicates less favorable conditions for water adsorption, i.e., similar sorption energies of additional water molecules after chemisorption, because of the larger pore diameter and hydrophobic moieties. Therefore, water cluster formation is the driving force for pore condensation, initiated by previously adsorbed water molecules coordinating to the metal centers [56,57]. After this step, more water molecules are gradually adsorbed over a large pressure range. Desorption proceeds gradually as no pronounced desorption steps are visible. This results in a hysteresis between the first adsorption and desorption branch, again suggesting the formation of a meta-stable state during the adsorption process. The total water uptake after the first adsorption branch (0.43 ± 0.03 g g^−1^) is slightly smaller than for Ni_2_dhtp, which is attributed to the higher molecular mass of the used dondc^4-^ ligand, resulting in a larger molecular mass. However, our results are in agreement with single crystal data on as-synthesized Mn_2_dondc [20], suggesting that, once completely hydrated, water occupies similar positions in fully hydrated *M*_2_dhtp (i.e., five distinct positions) (We note that the authors mentioned significant disorder of the water molecules due to residual *N*-Methyl-2-pyrolidone solvent molecules). However, unlike for Ni_2_dhtp, these distinct positions do not stem from the occupation of preferred sorption sites during the adsorption but rather from an attractive configuration of adsorbed water molecules in hydrated pores. Continuing with the second cycle, a lower overall uptake is observed after the adsorption branch, suggesting decomposition of the framework during the measurement, applying a similar mechanism as discussed for Ni_2_dhtp [42,46,47], i.e., with partial pore blockage, resulting in decreased water uptake.

**Figure 6:**
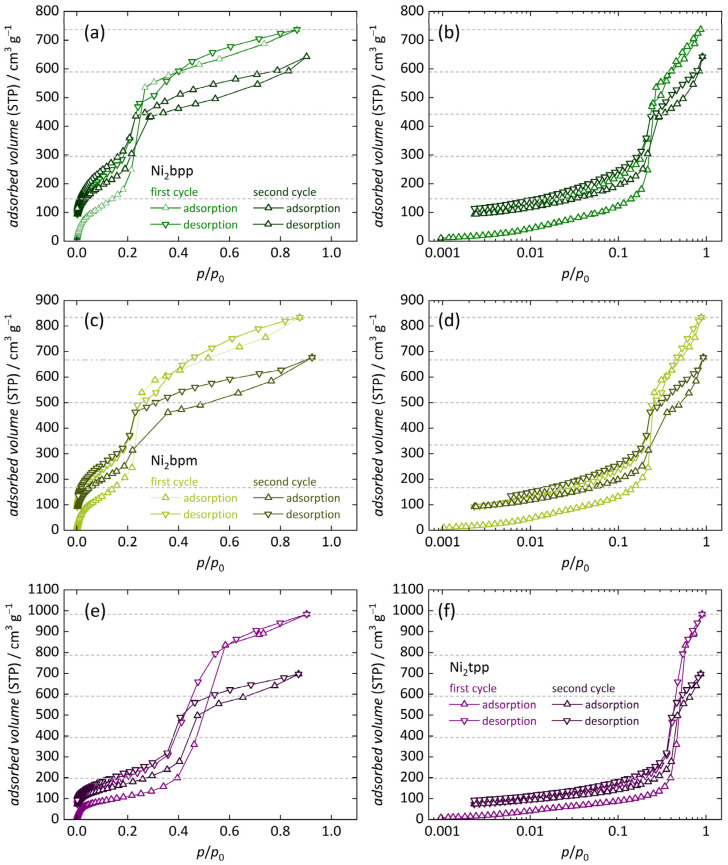
Water vapor sorption isotherms (25 °C) of Ni_2_bpp, Ni_2_bpm and Ni_2_tpp (two consecutive adsorption/desorption cycles). Data are shown at linear scale (**a**,**c**,**e**) and in semi-logarithmic representation (**b**,**d**,**f**). Horizontal lines mark 20%, 40%, 60%, 80% and 100% of the total water uptake during the first cycle (Missing data points for Ni_2_bpm below ca. *p*/*p*_0_ = 0.006 stem from technical difficulties).

Finally, we investigated the adsorption/desorption behavior of the remaining Ni_2_*L* materials (i.e., *L* = bpp, bpm and tpp). These compounds offer even larger pores than Ni_2_dondc, but reveal distinct similarities, showing type IV [54] water sorption isotherms. All these Ni_2_*L* materials reveal only minor adsorption below a pressure of *p*/*p*_0_ < 0.2, which is again attributed to the irreversible adsorption to the primary sorption sites (Ni^2+^ metal sites, Figure 6a–f). The amount of irreversibly bond water molecules decreases as shown by 13% (Ni_2_bpp) > 11% (Ni_2_bpm) > 8% (Ni_2_tpp), suggesting a smaller amount of water strongly interacting with the framework, resulting in a more bulk-like state, agreeing with our FTIR experiments. Slight differences in the trend presumably stem from the significantly higher incorporation of For^−^ within Ni_2_bpp, resulting in lower hydrolytic stability. As the relative water pressure increases, one large adsorption step is visible in all isotherms, which is shifted toward higher pressures with increasing pore size: *p*/*p*_0_ ≈ 0.21 for (Ni_2_bpp and Ni_2_bpm) < *p*/*p*_0_ ≈ 0.46 for (Ni_2_tpp). Similar dependencies of the condensation of water on the pore size were reported for other MOFs [10] and carbon nanotubes [81,87,88]. These pronounced sorption steps are accountable for the majority of the total water uptake, indicating that for Ni_2_dondc and other MOFs [56,57], water adsorption is driven by water cluster formation initiated by chemisorbed water molecules, facilitating pore condensation. Over the remaining pressure ranges, water is gradually adsorbed until complete hydration is observed. During desorption, a small hysteresis is observed, attributed to the above-stated preliminary pore condensation. However, a rather unusual behavior is found for Ni_2_bpp and Ni_2_bpm, as the first desorption branch crosses the first adsorption branch within the pressure range *p*/*p*_0_ = 0.25 … 0.41 (Figure 6a–d). This is assigned to structural degradation of the framework [89], adopting a similar decomposition mechanism as reported for Ni_2_dhtp [42,47]. There, additional open metal sites are formed with individual pores tilting against each other, resulting in decreased porosity. Further evidence for this is found as the second adsorption/desorption cycle is considered, revealing a significantly reduced water uptake after the second adsorption branch when compared to the first adsorption branch. We note that a similar phenomenon might occur for Ni_2_tpp (see data range from *p*/*p*_0_ = 0.5 … 0.7) although data are inconclusive in this respect (Figure 6e,f). In addition to this crossing, the second cycles are mostly identical to the first ones, clearly indicating that this effect is caused by framework degradation rather than unique material properties. The maximum water uptake after the first sorption branch increases with pore size, 0.57 ± 0.02 g g^−1^ (Ni_2_bpp) < 0.65 ± 0.07 g g^−1^ (Ni_2_bpm) < 0.80 ± 0.01 g g^−1^ (Ni_2_tpp). Except for the observed crossing of the branches, our results on Ni_2_bpp and Ni_2_tpp are generally in agreement with previous studies, revealing a slightly lower total water uptake [17]. Differences are attributed to either different measurement settings (fewer data points in the respective crossing regions) or lower defect amounts (not analyzed therein).

**Figure 7:**
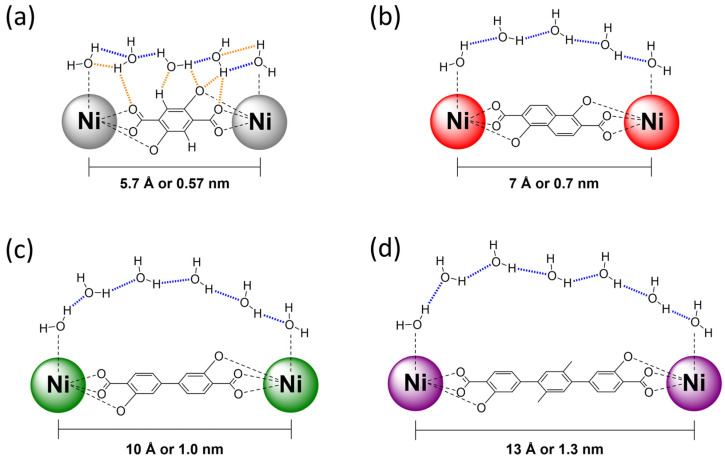
Illustration of the different arrangements of water molecules near the framework pore walls in hydrated Ni_2_*L* frameworks. (**a**) Ni_2_dhtp reveals multiple attractive H-bonding interactions with framework atoms (dashed orange lines) and surrounding water molecules (dashed blue lines), located at distinct positions. Formed water bridges, avoiding the hydrophobic domains in (**b**) Ni_2_dondc, (**c**) Ni_2_bpp and (**d**) Ni_2_tpp, suggest that the size of the water bridges/chains increases with larger distances of adjacent coordinating water molecules, i.e., H-bonding anchors (distances estimated from XRD data [11,20,97]).

When compared to our TGA results, the overall water uptake determined by water vapor sorption experiments (see Supplementary Materials Table S4) is higher for all frameworks (23 … 48 mmol g^−1^). Differences become larger with increasing pore size. As stated above, our TGA analysis underestimates the amount of adsorbed water because the chemisorbed water is strongly bound and gradually released over a larger temperature range and during decomposition. Hence, it is not considered in the initial dehydration step. While this is sufficient to explain the difference for Ni_2_*L* frameworks with a smaller pore apparatus, i.e., *L* = dhtp, dhip and dondc, additional factors for larger pores must be considered. We hypothesize that some water molecules in these frameworks, i.e., *L* = bpp, bpm, tpp, are loosely bond and could leave prior to the TGA measurements during sample preparation. Further, we note that we do not see a clear trend of the maximum adsorbed volumes in our nitrogen sorption and water sorption experiments (see Table S6), as found in other studies [82]. Nevertheless, our water vapor sorption analysis indicates that additional hydrophilic sorption sites for H-bonding, besides the open metal sites, i.e., near the inorganic SBUs as present in Ni_2_dhtp, are optimal for high water sorption at low relative pressures (Figure 7a). Those additional sorption sites are not found in the other members of the Ni_2_*L* series, which is an immediate consequence of the larger hydrophobic domains. We suggest that water adsorption within the more porous Ni_2_*L* frameworks (*L* = dondc, bpp, bpm, tpp), is dominated by water cluster formation and follows a similar mechanism in the low-pressure region, as previously reported for other MOFs with strong sorption sites [56,57,58]. First, the open metal sites are saturated before additional, low-entropy water molecules adsorb thereon, forming distinct water clusters driven by large hydration enthalpies (We note that this could also be a simultaneous process). With increasing pressures, previously adsorbed water molecules initiate the formation of water bridges/chains between adjacent chemisorbed water molecules, acting as hydrophilic anchor sites (Figure 7b–d), similar to studies previously reported for mesoporous silica [83,90] and porous carbons [91] possessing similar hydrophobic domain size. As soon as enough water molecules are present in the gas phase to connect enough adjacent anchor sites, the formation of a continuous H-bonding network is initiated, resulting in pore condensation with minimal framework–water interactions. This process is shifted toward higher relative pressures *p*/*p*_0_ with increasing hydrophobic domain size, i.e., larger distances of adjacent chemisorbed water molecules, revealing a direct correlation of the inflection point *α* and the linker polarity (i.e., octanol–water partition coefficients [92,93], see Table S5). Further, this suggests that the pore wall surface polarity is mostly controlled by the inorganic domains, even at small domain sizes. These effects are a direct consequence of the Gibbs hydration energy Δ*G*_hydration_ being dominated by the enthalpy term for larger hydrophobic domains, with water molecules avoiding these units. Conversely, smaller units are viewed as discrete perturbations, forcing a high-entropy water arrangement near the hydrophobic surface, resulting in dominant entropic contributions as suggested for Ni_2_dhtp (i.e., dominant surface–water interactions) [94,95,96]. The transition length for the reversal of entropy/enthalpy-driven hydration lies around 10 Å (or 1 nm), which is within the same range as water–water distances for coordination water molecules in Ni_2_bpp and Ni_2_bpm. Following the condensation step, the relaxation of adsorbed water molecules provides additional room for further water molecules until complete hydration is observed. Consequently, a more bulk-like water arrangement with increased H-bonding symmetry is found, as suggested by our FTIR experiments. Hence, the observed H-bonding in NW water is dominated by water–water interactions for larger pores, which is additionally supported by the observation of a decreasing amount of irreversible bond water molecules with increasing pore size. For Ni_2_dondc, this relaxation process initiates the formation of more entropic water molecules with more asymmetric H-bonding forced to interact with the pore walls. Eventually, this results in the occupation of discrete positions, as suggested by XRD data [20], even though the initial hydration mechanism, i.e., pore condensation, is enthalpy-driven. This is supported by our spectroscopic results, highlighting a more asymmetric H-bonding situation.

## 4. Conclusions

Our study on the isoreticular Ni_2_*L* framework series enabled us to analyze the effects of the pore size and linker hydrophobicity on the water sorption mechanism and the arrangement of confined water. We found evidence that defects in Ni_2_dhtp play an important role on the given sorption mechanism. Higher amounts of formate defects result in a less uniform pore wall, reducing the attractiveness of the third sorption site (near the phenylene backbone), offering less H-bonding possibilities. Conversely, incorporation of fewer defect sites results in a sorption mechanism, more comparable to those discussed for most other *M*_2_dhtp frameworks. This suggests that selective incorporation of defects enables one to precisely tune the water sorption properties in the *M*_2_dhtp series. On the other hand, these effects play a minor role for larger pores (i.e., Ni_2_*L*; *L* = dondc, bpp, bpm, tpp). Within the low-pressure range, water predominantly adsorbs to the open metal sites while additional molecules adsorb thereon as a result of large hydration enthalpies, forming distinct water clusters. Water bridges between adjacent chemisorbed water molecules, located at the primary binding sites, form thereafter, providing additional H-bonding opportunities. Consequently, the sorption process is facilitated, resulting in classical pore condensation characterized by a continuous H-bonding network. The observed sorption step is governed by the pore size, with the pressure range additionally depending on the linker hydrophobicity/hydrophobic domain size. We found that H-bonding is generally more asymmetric in smaller pores, consistent with the expectation of defined water positions in fully hydrated Ni_2_dhtp and Ni_2_dondc, offering multiple attractive water–framework interactions. Larger pores offer a more symmetric H-bonding situation, suggesting a more bulk-like water arrangement under less confinement, dominated by water–water interactions, as indicated by decreasing dehydration temperatures with increasing pore size.

## Figures and Tables

**Table 1:** Determined lattice plane distance *d*_110_ calculated from the Bragg equitation as well as surface areas *S*_BET_, pore volumes *V*_Pore_ and pore sizes *d*_Pore,NLDFT_ of Ni_2_*L* materials determined from nitrogen sorption data at 77 K.

	*d*_110_ (Å)	*S*_BET_ (m^2^ g^−1^)	*V*_Pore_ (cm^3^ g^−1^)	*d*_Pore,NLDFT_ (Å)
Ni_2_dhtp	12.93 ± 0.02	1240 ± 80	0.55 ± 0.07	12.73 ± 0.22
Ni_2_dhip	12.97 ± 0.06	560 ± 120	0.38 ± 0.09	11.93 ± 0.30
Ni_2_dondc	14.57 ± 0.10	1010 ± 10	0.55 ± 0.03	13.63 ± 0.42
Ni_2_bpp	18.36 ± 0.04	1660 ± 230	0.74 ± 0.02	16.68 ± 0.57
Ni_2_bpm	18.87 ± 0.00	1200 ± 50	0.58 ± 0.02	18.52 ± 0.52
Ni_2_tpp	23.05 ± 0.06	2320 ± 140	1.03 ± 0.06	25.09 ± 0.67

**Table 2:** Determined linker and formate (For^−^) or acetate (OAc^−^) capping ligand concentrations of Ni_2_*L* materials derived from solution ^1^H NMR and their respective sum formulas, considering charge balance. (Sum formula for a defect free material would be Ni_2_*L*.).

	Linker Molecules per Formula Unit	For^−^/OAc^−^ Ligands per Formula Unit	Sum Formula
Ni_2_dhtp	0.84 ± 0.02	0.62 ± 0.10	Ni_2_(dhtp)_0.84_(For)_0.62_
Ni_2_dhip	0.65 ± 0.09	1.41 ± 0.35	Ni_2_(dhip)_0.65_(For)_1.41_
Ni_2_dondc	0.65 ± 0.01	1.39 ± 0.00	Ni_2_(dondc)_0.65_(OAc)_1.39_
Ni_2_bpp	0.49 ± 0.06	2.04 ± 0.25	Ni_2_(bpp)_0.49_(For)_2.04_
Ni_2_bpm	0.61 ± 0.06	1.54 ± 0.25	Ni_2_(bpm)_0.61_(For)_1.54_
Ni_2_tpp	0.54 ± 0.04	1.85 ± 0.16	Ni_2_(tpp)_0.54_(For)_1.85_

**Table 3:** Wavenumber splitting Δ$\tilde{v}$ of the stretching vibration bands of water in the Ni_2_*L*-hyd materials and of pure water.

	Δ$\tilde{v}$_multimer-network_ (cm^−1^)	Δ$\tilde{v}$_multimer-intermed._ (cm^−1^)	Δ$\tilde{v}$_network-intermed._ (cm^−1^)
Ni_2_dhtp	336.2 ± 2.5	146.1 ± 1.1	190.1 ± 2.5
Ni_2_dhip	325.4 ± 1.8	142.2 ± 1.2	183.2 ± 1.8
Ni_2_dondc	337.0 ± 2.5	141.3 ± 1.2	181.7 ± 1.7
Ni_2_bpp	321.0 ± 1.9	140.9 ± 1.5	182.9 ± 1.6
Ni_2_bpm	320.6 ± 1.7	142.6 ± 1.4	178.8 ± 1.5
Ni_2_tpp	309.1 ± 2.2	140.0 ± 2.0	169.1 ± 1.8
water	284.4 ± 1.5	130.2 ± 2.9	154.2 ± 1.5

## Data Availability

Dataset available on request from the authors.

## Supplementary Materials

The following supporting information can be downloaded at: https://www.mdpi.com/article/10.3390/nano14221791/s1, Synthetic procedures (linker and MOF synthesis), Figures S1–S5: NMR spectra of organic compounds; Figures S6–S11: Pore size distribution fits by NLDFT methods; Figures S12–S18: NMR spectra of digested MOFs; Figures S19–S24: FTIR spectra of MOFs in comparison with the used linkers; Figure S25: Deconvolution of the O-H stretching vibration of liquid water; Figure S26: XRD data of re-isolated MOF samples after water vapor sorption analysis; Figures S27–S32: Normalized first derivatives (δ*V*/δ(*p*/*p*_0_)) of water vapor sorption data; Table S1: Total water uptake of MOF samples, determined by TGA analysis; Table S2: Results of least square fits of hydrated MOF samples in comparison with pure water; Table S3: Peak center position of the O-H stretching vibration of water in hydrated MOFs; Table S4: Total water uptake of MOF samples, determined by water vapor sorption analysis; Table S5: Octanol–water partition coefficients of protonated linker molecules; Table S6: Comparison of the maximum adsorption of the nitrogen and water sorption isotherms.
