# Supplementary material for: Water Sorption on Isoreticular CPO-27-Type MOFs: From Discrete Sorption Sites to Water-Bridge-Mediated Pore Condensation
Source: Nanomaterials (Basel). 2024 Nov 7;14(22):1791. doi: 10.3390/nano14221791 (PMC11597837; doi:10.3390/nano14221791)
Supplement: Supplementary file 1 [file nanomaterials-14-01791-s001.zip › nanomaterials-3239837-supplementary.pdf]

Water Sorption Studies on Isoreticular CPO-27-type MOFs: From Discrete Sorption Sites to Water-Bridge-Mediated Pore Condensation

Supporting Information

Marvin Kloß, Lara Schäfers, Zhenyu Zhao, Christian Weinberger, Hans Egold and Michael Tiemann*

Department of Chemistry, Faculty of Science, Paderborn University
Warburger Str. 100 D-33098, Paderborn, Germany

Inorganic Chemistry (michael.tiemann@upb.de)

EXPERIMENTAL (LINKER SYNTHESIS)

Synthesis of 1,5-dihydroxynaphthalene-2,6-dicarboxylic acid (H_4dondc): Synthesis was performed using a previously published protocol under slight modifications.[1] 1,5-dihydroxynaphtalene (9.75 g, 60 mmol, 1.0 eq) and potassium bicarbonate (20.25 g, 202.5 mmol, 3.375 eq) were finely grounded in a mortar. The solid mixture was transferred to a Teflon-lined inlay (125 mL), placed in a stainless-steel reactor (*Parr Instruments*), sealed, and reacted in a pre-heated oven at 230 °C for 20 h. After cooling to room temperature, the mixture was carefully acidified using aqueous hydrochloric acid (2 M). The solid was separated by centrifugation (4000 rpm, 30 min) and washed three time with desalinated water. The obtained green solid residue was dried at 60 °C over the course of three days before it was re-dissolved in tetrahydrofuran (THF) and subsequently filtered to get rid of solid impurities. Then, THF was removed under reduced pressure, yielding a brown solid. Finally, dispersion of the solid in fresh methanol and removal of the solvent under reduced pressure yielded 1,5-dihydroxynaphthalene-2,6-dicarboxylic acid (H_4dondc) as a green-brown powder (13.28 g, 53.5 mmol, 88%).

^{1}H NMR (500 MHz, dmso-d$_6$): δ = 14.02 (brs, 2H), 12.7 (brs, 2H), 7.81 (d, 2H, $^3J_{HH}$ = 8.8 Hz), 7.74 (d, 2H, $^3J_{HH}$ = 8.8 Hz) ppm.

^{13}C NMR (125 MHz, dmso-d$_6$) δ = 172.5, 159.3, 127.6, 124.9, 113.1, 109.0 ppm (see **Figure S1**).

Synthesis of 3,3'-dihydroxy-[1,1'-biphenyl]-4,4'-dicarboxylic acid (H_4bpp): For the first step, a Suzuki-Miyaura cross coupling reaction was performed following a literature protocol with slight modifications.[2] Potassium acetate (2.52 g, 25.71 mmol, 6.3 eq), and Pd(dppf)Cl_2·DCM (200 mg, 0.245 mmol, 0.06 eq) were placed in a Schlenk-flask (100 mL) inside an argon glovebox. Then, 3-methoxy-4-methoxycarbonylphenyl boronic acid (1.03 g, 4.9 mmol, 1.2 eq) and methyl-4-bromo-2-methoxybenzoat (1.0 g, 4.08 mmol, 1.0 eq) were added under a constant argon gas flow. All compounds were suspended in a solvent mixture of *p*-dioxane/water (25/10 ml, previously degassed for 45 min). The mixture was heated to 80 °C for 19 h. After cooling to room temperature, the solid residue was filtrated and washed with water (total of 200 mL in several portions). Subsequent drying for 48 h at 60 °C in an oven yielded dimethyl-3,3′-dimethoxy-[1,1′-biphenyl]-4,4′-dicarboxylate as a brown solid (1.3 g, 3.94 mmol, 96%).

^{1}H NMR (500 MHz, $CDCl_3$): δ=7.89 (d, $^3J_{HH}$ = 8 Hz, 1H), 7.20 (dd, $^3J_{HH}$ = 8 Hz, $^4J_{HH}$ =1.5Hz, 1H), 7,15 (d, $^4J_{HH}$ = 1.5Hz, 1H), 3.98 (s, 6H), 3.92 (s, 6H) ppm. (see **Figure S2**).

For the second step, two subsequent deprotection reactions were performed with slight deviation from previously published protocols.[3] Dimethyl-3,3′-dimethoxy-[1,1′-biphenyl]-4,4′-dicarboxylate (1.3 g, 3.94 mmol, 1 eq) was dissolved in dry dichloromethane (DCM, 15mL). The solution was cooled to –78 °C using an acetone cooling bath. Then, boron tribromide solution, 1 M in *n*-hexane, (13 mL, 13 mmol, 3.3 eq) was slowly added with a syringe. After complete addition, the mixture was cooled for additional 5 min before removal of the cooling bath. The mixture was stirred over night at room temperature. The reaction was

quenched using ice cold water (same volume than DCM). The solid residue was isolated by filtration and dried at 60 °C overnight. Then, the filtrate was saturated with sodium chloride and extracted with ethyl acetate (3 x 50 mL). Combined organic layers were dried over $MgSO_4$ and the solvent was removed under reduced pressure. The obtained solid was then dried at 60 °C overnight. For the next step, both crude products were combined (1.41 g, 4.67 mmol, *ca.* 1 eq) and dissolved in a mixture of THF (12 mL) and aqueous sodium hydroxide (1.87 g, 46.7 mmol, 10 eq, 12 mL) to obtain a 0.2 M solution. Mixture was heated to 50 °C for 24h. After cooling to room temperature, THF was removed under reduced pressure. Next, aqueous hydrochloric acid was added to the suspension until a pH < 2 was reached. The precipitate was filtered, washed with copious amounts of water, and dried for 48 h at 60 °C. 3,3′-Dihydroxy-[1,1′-biphenyl]-4,4′-dicarboxylic acid (H_4bpp) was isolated as an off-white solid (0.91, 3.0 mmol, 76%).

^{1}H NMR (500 MHz, dmso-d$_6$): δ= 11.41 (brs, 2H), 7.87 (d, $^3J_{HH}$ = 8.0 Hz, 1H), 7.20 (dd, $^3J_{HH}$ = 8.0 Hz, $^4J_{HH}$ =1.5Hz, 1H), 7,15 (d, $^4J_{HH}$ =1.5Hz, 1H), 3.98 (s, 6H), 3.92 (s, 6H) ppm.

^{13}C NMR (125 MHz, dmso-d$_6$) δ = 166.5, 159.7, 145.8, 132.5, 119.8, 119.4, 111.3, 56.4, 52.2 ppm (see **Figure S3**).

Synthesis of 3,3''-dihydroxy-2',5'-dimethyl-[1,1':4',1''-terphenyl]-4,4''-dicarboxylic acid (H_4tpp):

For the first step, a Suzuki-Miyaura cross coupling reaction was performed following a literature protocol under slight modifications.[2] Potassium acetate (2.23 g, 22.73 mmol, 12 eq), and Pd(dppf)Cl_2·DCM (155 mg, 0.189 mmol, 0.1 eq) were placed in a Schlenk-flask (50 mL) inside an argon glovebox. Then, 3-methoxy-4-methoxycarbonylphenyl boronic acid (0.955 g, 4.55 mmol, 2.4 eq) and 1,4-dibromo-2,5-dimethylbenzene (0.5 g, 1.89 mmol, 1.0 eq) were added under a constant argon gas flow. All compounds were suspended in a solvent mixture of *p*-dioxane/water (25/10 ml, previously degassed for 45 min). The mixture was refluxed overnight (*ca.* 16 h). After cooling to room temperature, water (20 mL) was added, and the aqueous phase was extracted with DCM (3 x 25ml). Combined organic phases were dried over $MgSO_4$. Removing of the solvent under reduced pressure and subsequent drying at 60 °C over 48 h yielded dimethyl-3,3′-dimethoxy-2,5-dimethyl-[1,4-terphenyl]-4,4′-dicarboxylate as a off-white solid (0.55 g, 1.22 mmol, 67%).

^{1}H-NMR δ=7.86 (d, $^3J_{HH}$ = 7.8 Hz, 2H), 7.16 (s, 2H), 6.98 (dd, $^3J_{HH}$ = 7.8 Hz, $^4J_{HH}$ = 1.4 Hz, 2H), 6.96 (d, $^4J_{HH}$ = 1.4 Hz, 2H), 3.93 (s, 6H), 3.92 (s, 3H), 2.28 (s, 6H) ppm (see **Figure S4**).

For the second step, two subsequent deprotection reactions were performed with slight deviation from previously published protocols.[3] Dimethyl-3,3′-dimethoxy-2,5-dimethyl-[1,4-terphenyl]-4,4′-dicarboxylat (1.14 g, 2.62 mmol, 1 eq) was dissolved in dry DCM (15 mL). The solution was cooled to -78 C using an acetone cooling bath. Then, boron tribromide solution, 1 M in *n*-hexane, (7.8 mL, 7.8 mmol, 3.3 eq) was slowly added with a syringe. After complete addition, the mixture was cooled for additional 5 min before removal of the cooling bath. The mixture was stirred over night at room temperature (*ca.* 16 h). The reaction was quenched

using ice cold water (same volume than DCM). The solid residue was isolated by filtration and dried at 60 °C overnight. Then, the filtrate was saturated with sodium chloride and extracted with ethyl acetate (3 x 50 mL). Combined organic layers dried over $MgSO_4$ and the solvent was removed under reduced pressure. The obtained solid was then dried at 60 °C overnight. For the next step, both crude products were combined (0.754 g, 1.86 mmol, 1 eq) and dissolved in a mixture of THF (5 mL) and aqueous sodium hydroxide (0.742 g, 18.6 mmol, 10 eq, 5 mL) to obtain a 0.2 M solution. The mixture was heated to 50 °C for 24h. After cooling to room temperature, THF was removed under reduced pressure. Aqueous hydrochloric acid was added to the suspension until a pH <2 was reached. The precipitate was filtered, washed with copious amounts of water, and dried for 48 h at 60 °C. 3,3′-Dihydroxy-[1,1′-biphenyl]-4,4′-dicarboxylic acid (H_4tpp) was isolated as an off-whit solid (0.91, 1.65 mmol, 91 %).

^{1}H NMR (700 MHz, $CDCl_3$): δ= 7.85 (d, $^3J_{HH}$ = 8.4 Hz, 2H), 7.17 (s, 2H), 6.93 (dd, $^3J_{HH}$ = 8.4 Hz, $^4J_{HH}$ = 1.5 Hz, 1H), 6.92 (d, $^4J_{HH}$ =1.5 Hz, 2H), 2.24 (s, 6H) ppm.

^{13}C NMR (175 MHz, dmso-d_6) δ = 171.8, 160.9, 148.2, 139.6, 132.2, 131.3, 130.1, 120.2, 117.3, 111.7, 19.5 ppm (see **Figure S5**).

EXPERIMENTAL (MOF SYNTHESIS)

Preparation of Ni_2(dhtp)[4]: H_4dhtp (349 mg, 1.76 mmol, 0.6 eq) was dissolved in *N,N*-dimethylformamide (DMF, 50 mL) using ultra sonification. Separately, nickel(II) nitrate hexahydrate (854 mg, 2.94 mmol, 1.0 eq) was dissolved in DMF (12.5 mL) and deionized water (3.2 mL). Both solutions were combined in a Teflon-lined insert (125 mL) and the mixture was stirred briefly. The insert was placed in a stainless-steel reactor (*Parr Instruments*), sealed, and reacted in a preheated oven at 100 °C for 24 h. After cooling, the solid was separated by filtration and washed with DMF (2 x 20 mL) deionized water (3 x 30 mL) and methanol (3 x 30 mL). Then, the solvent was exchanged using methanol (three times over the course of four days). The obtained product was dried in multiple steps. For the first step, the product was placed in a round flask under dynamic vacuum for 16 h. Then, the product was heated in 70 °C (hold for 18 h). The desolvated product (orange) was stored in a glove box under argon atmosphere.

Preparation of Ni_2(dhip)[5]: H_4dhip (149 mg, 0.75 mmol, 1.0 eq) was dissolved in DMF (12.5 mL) using ultra sonification. Separately, nickel(II) chloride hexahydrate (446 mg, 1.88 mmol, 2.5 eq) was dissolved in a mixture of DMF (20 mL) and methanol (17.5 mL). Both solutions were portioned equally to five screw-top jars (20 mL, with PTFE seal). The sealed jars were heated to 120 °C for 18 h in a pre-heated oven. After cooling to room temperature, the solid residues were combined and washed with DMF (3 x 25 mL) and methanol (3 x 25 mL). Then, the solvent was exchanged using methanol (four times *ca.* 15mL over the course of four days). The obtained product was dried in multiple steps. For the first step, the product was placed in a round flask under dynamic vacuum for 16 h. Then, the product was heated to 70 °C (hold for 18 h). The desolvated product (light-green) was stored in a glove box under argon atmosphere.

Preparation of Ni_2(dondc)[1]: H_4dondc (310 mg, 1.25 mmol, 1.0 eq) was dissolved in *N*-Methyl-2-pyrrolidon (NMP, 40 mL) using ultra sonification. Separately, nickel(II) acetate tetrahydrate (620 mg, 2.5 mmol, 2.0 eq) were dissolved in deionized water (5 mL). Both solutions were portioned equally to five screw-top jars (20 mL, with PTFE seal). The sealed jars were heated to 110 °C for 48 h in a pre-heated oven. After cooling to room temperature, the solid residues were combined and washed with DMF (3 x 25 mL), and methanol (3 x 25 mL). Then, the solvent was exchanged using methanol (three times 15 mL over the course of four days). The obtained product was dried in multiple steps. For the first step, the product was placed in a round flask under dynamic vacuum for 16 h. Then, the product was heated in 70 °C (hold for 18 h). The desolvated product (brown) was stored in a glove box under argon atmosphere.

Preparation of Ni_2(bpp): Synthesis was performed by upscaling of a previously reported procedure.[6] H_4bpp (153 mg, 0.57 mmol, 1.0 eq) was dissolved in DMF (39 mL). Separately, nickel(II) nitrate hexahydrate (540 mg, 1.86 mmol, 3.3 eq) was dissolved in DMF (6 mL). Both solutions were transferred to a Teflon-lined insert (125 mL). Then, ethanol (1 mL) and deionized water (1 mL) were added. The insert was placed in a stainless-steel reactor (*Parr Instruments*), sealed, and reacted in a preheated oven at 120 °C for 24 h. After cooling to room temperature, the overstanding solution was separated, and the solid residue was dispersed in DMF (15 mL). The solvent was replaced four-times over the course of several days. Then, the solvent was exchanged using methanol (2 x 20mL over the course of four days). The obtained product was dried in multiple steps. For the first step, the product was placed in a round flask under dynamic vacuum for 16 h. Then, the product was heated in 70 °C (hold for 18 h). The desolvated product (light-green) was stored in a glove box under argon atmosphere.

Preparation of Ni_2(bpm): Synthesis was performed by upscaling of a previously reported procedure under use of the H_4bpm linker molecule.[6] H_4bpm (156 mg, 0.57mmol, 1.0 eq) was dissolved in DMF (39 mL) using ultra sonification and transferred into a Teflon-lined inlay (125 ml). Separately, nickel(II) nitrate hexahydrate (540 mg, 1.86 mmol, 3.3 eq) was dissolved in DMF (6 mL) separately and added to the linker solution. Then, ethanol (3 mL) and deionized water (3 mL) were added, and the solution was briefly stirred. The inlay was transferred into a stainless-steel autoclave reactor (*Paar Instruments*), sealed, and heated to 120 °C for 24 h in a pre-heated oven. After cooling to room temperature, the solid residue was filtered of and washed with methanol (5 x 25 mL). Then, the solvent was exchanged using methanol (2 x 25mL over the course of four days). The obtained product was dried in multiple steps. For the first step, the product was placed in a round flask under dynamic vacuum for 16 h. Then, the product was heated in 70 °C (hold for 18 h). The desolvated product (light-green) was stored in a glove box under argon atmosphere.

Preparation of Ni_2(tpp): Synthesis is based on a previously published procedure.[6] H_4tpp (71 mg, 0.188 mmol, 1.0 eq) was dissolved in DMF (13 mL) in a screw-top jar with PTFE-sealed cap (20 mL) using ultra sonification. Separately, nickel(II) nitrate hexahydrate (180 mg, 0.62 mmol, 3.3 eq) was dissolved in DMF (2 mL) and added to the screw-top jar. Then, ethanol (1 mL) und deionized water (1 mL) were added. The jar war sealed, briefly shacked, and

heated to 120 C for 24 h in a pre-heated oven. After cooling to room temperature, the overstanding solution was separated, and the solid residue was dispersed in DMF (5 mL). The solvent was replaced four-times over the course of several days. Then, the solvent was exchanged using methanol (2 x 8mL over the course of four days). The obtained product was dried in multiple steps. For the first step, the product was placed in a round flask under dynamic vacuum for 16 h. Then, the product was heated in 70 °C (hold for 18 h). The desolvated product (brown-green) was stored in a glove box under argon atmosphere.

NMR (LINKER SYNTHESIS)

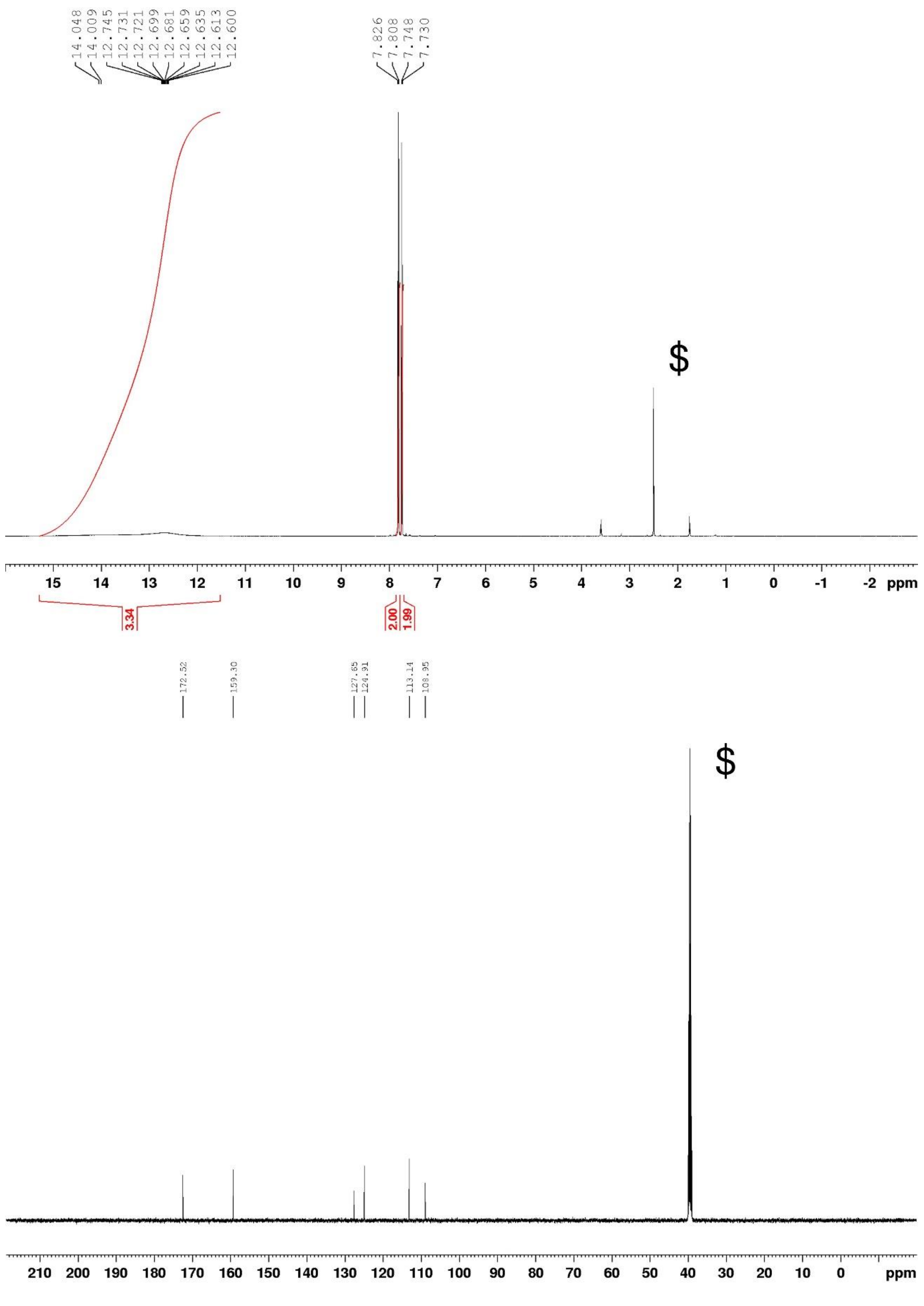

Figure S1. ^{1}H NMR (top) and ^{13}C NMR spectrum (bottom) of 1,5-dihydroxynaphthalene-2,6-dicarboxylic acid (H_4dondc) in dmso-d_6 (marked with $).

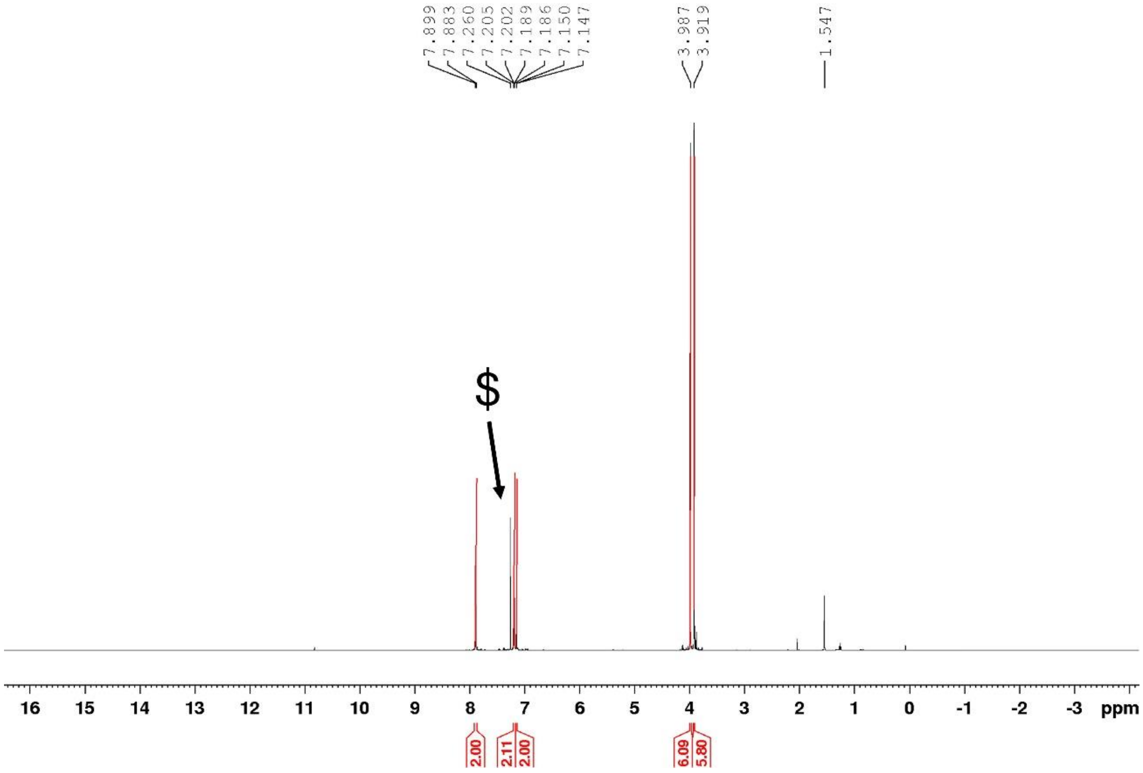

Figure S2. ^{1}H NMR spectrum of dimethyl-3,3'-dimethoxy-[1,1'-biphenyl]-4,4'-dicarboxylate in $CDCl_3$ (marked with $).

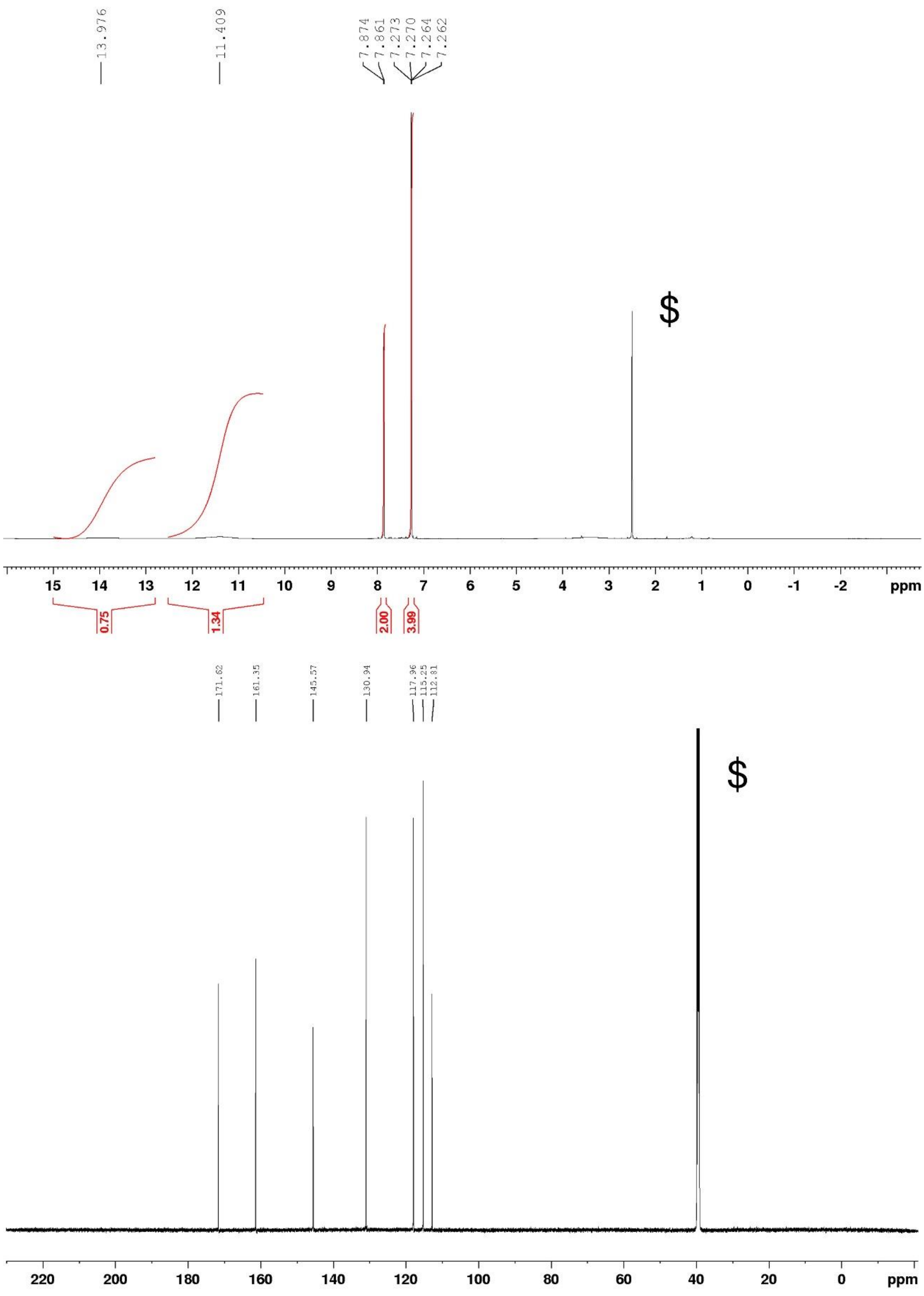

Figure S3. ^{1}H NMR (top) and ^{13}C NMR spectrum (bottom) of 3,3'-Dihydroxy-[1,1'-biphenyl]-4,4'-dicarboxylic acid (H_4bpp) in dmso-d_6 (marked with $).

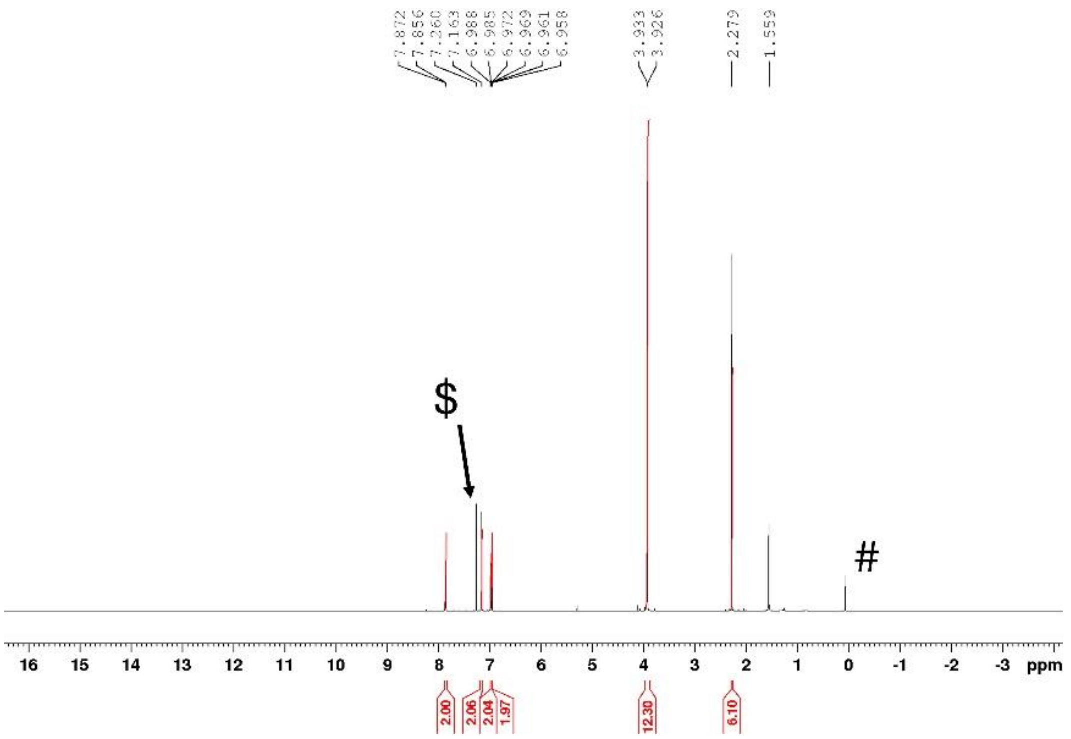

Figure S4. ^{1}H NMR spectrum of dimethyl-3,3′-dimethoxy-2,5-dimethyl-[1,4-terphenyl]-4,4′-dicarboxylate in $CDCl_3$ (marked with $). TMS (marked with #) has been used as internal standard.

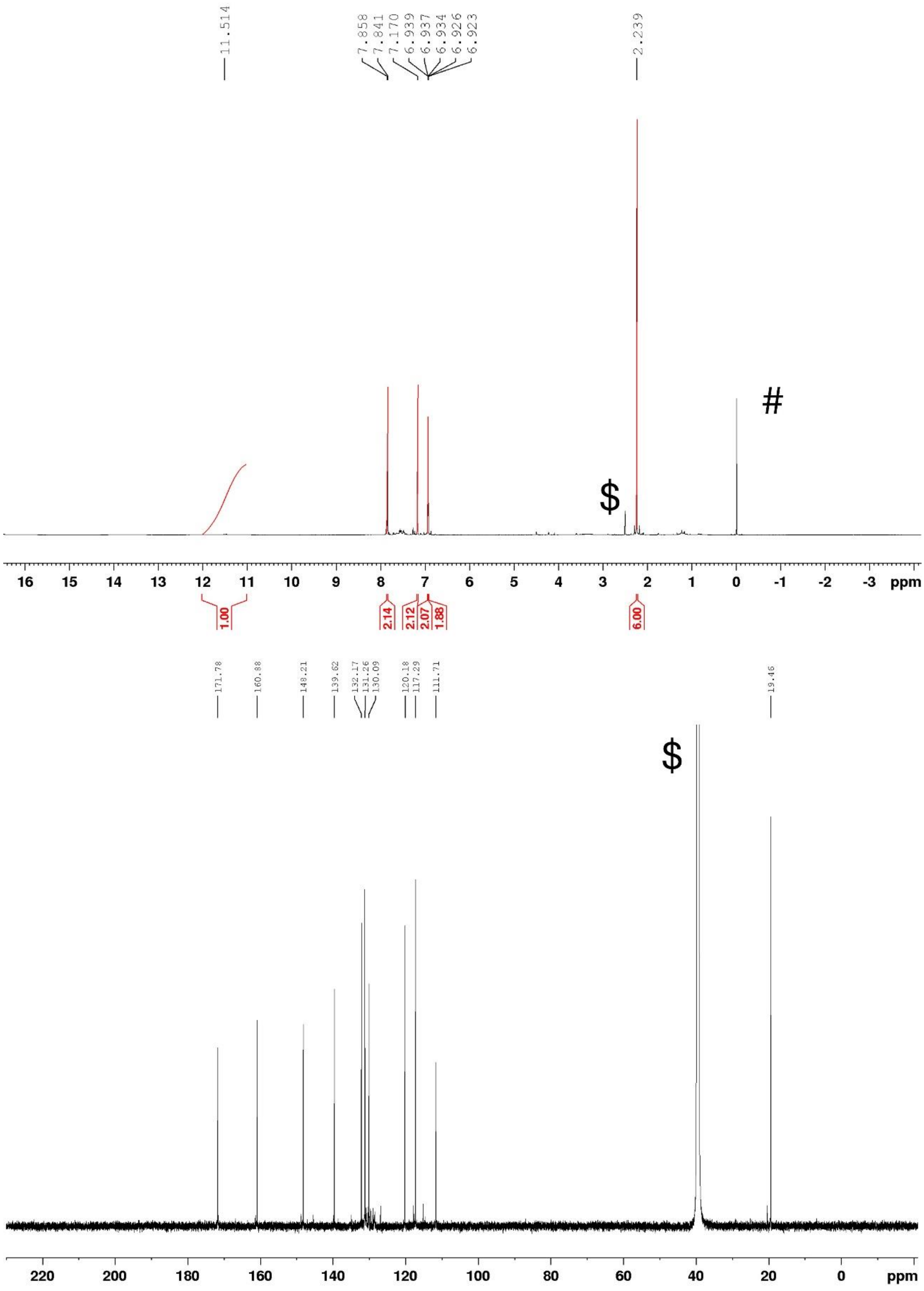

Figure S5. ^{1}H NMR (top) and ^{13}C NMR spectrum (bottom) of 3,3'-Dihydroxy-[1,1'-biphenyl]-4,4'-dicarboxylic acid (H_4tpp) in dmso-d_6 (marked with $). Internal standard: TMS (marked with #).

NITROGEN SORPTION DATA

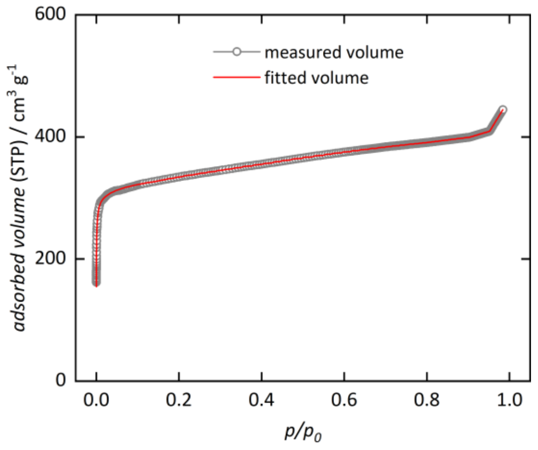

Figure S6. N_2 sorption data for Ni_2dhtp, obtained at 77 K, fitted by a NLDFT data-based method (Fitting error 0.2%).

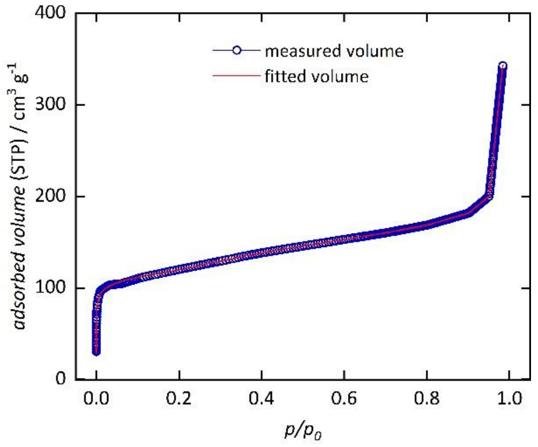

Figure S7. N_2 sorption data for Ni_2dhip, obtained at 77 K, fitted by a NLDFT data-based method (Fitting error 0.5%).

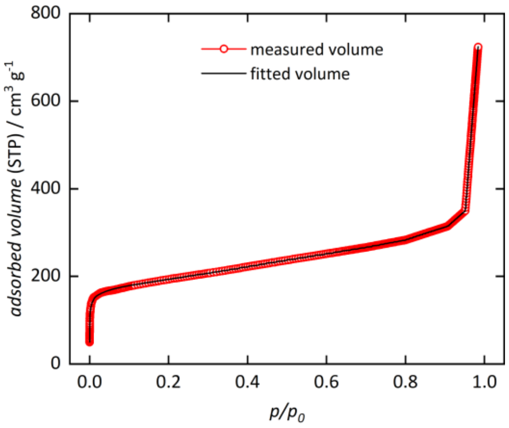

Figure S8. N_2 sorption data for Ni_2dondc, obtained at 77 K, fitted by a NLDFT data-based method (Fitting error 0.6%).

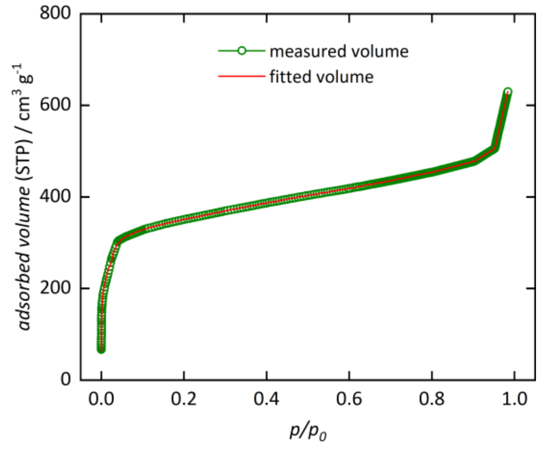

Figure S9. N_2 sorption data for Ni_2bpp, obtained at 77 K, fitted by a NLDFT data-based method (Fitting error 0.3%).

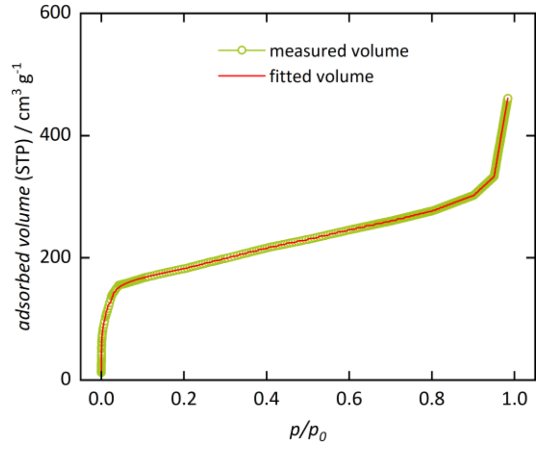

Figure S10. N_2 sorption data for Ni_2bpm, obtained at 77 K, fitted by a NLDFT data-based method (Fitting error 0.3%).

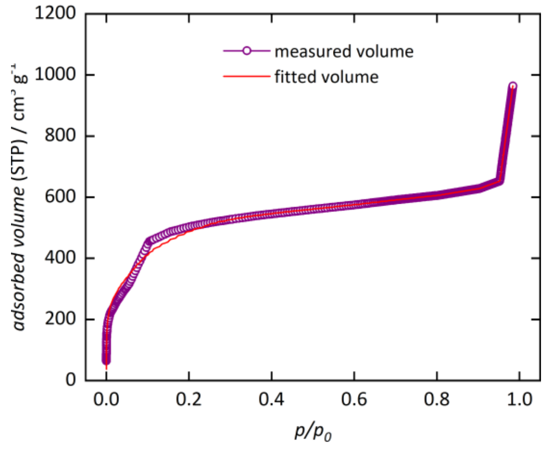

Figure S11. N_2 sorption data for Ni_2tpp, obtained at 77 K, fitted by a NLDFT data-based method (Fitting error 1.7%).

NMR (DIGESTED SAMPLES FOR DEFECT ANALYSIS)

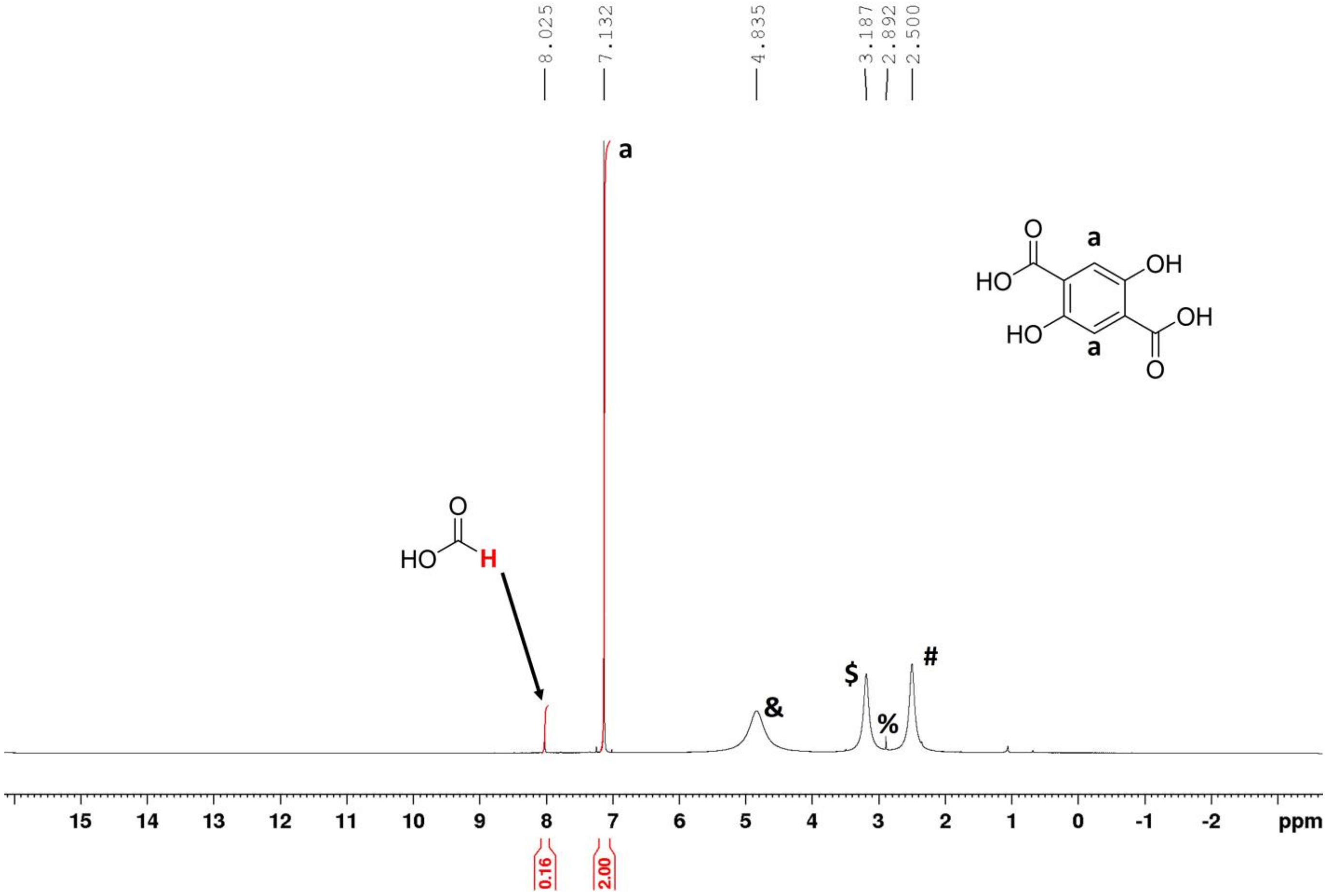

Figure S12. ^{1}H NMR Ni_2dhtp diluted in a mixture of dmso-d_6 (marked with #) and DCl (20wt% in D_2O, marked with $). The signal at 4.8 ppm is a superposition of all acidic protons (-OH and -COOH groups, marked with &), stemming from a fast chemical proton exchange. Residual DMF solvent molecules are marked with %. The remaining two DMF signals are not visible due to signal overlap or the low relative intensity.

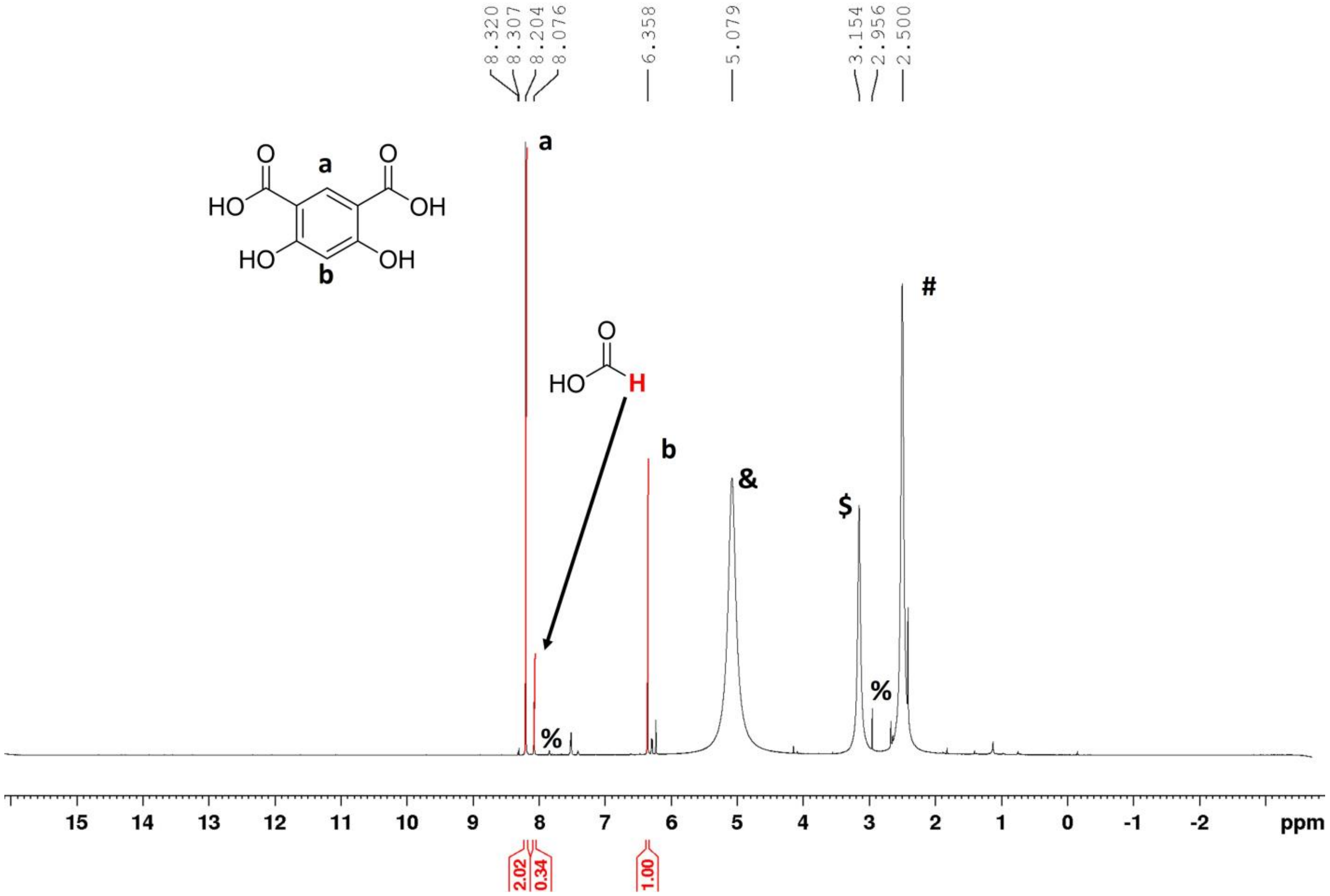

Figure S13. ^{1}H NMR Ni_2dhip diluted in a mixture of dmso-d_6 (marked with #) and DCl (20wt% in D_2O, marked with $). The signal at 4.8 ppm is a superposition of all acidic protons (-OH and -COOH groups, marked with &), stemming from a fast chemical proton exchange. Residual DMF solvent molecules are marked with %. The third DMF signal (methyl group) is not visible due to signal overlap.

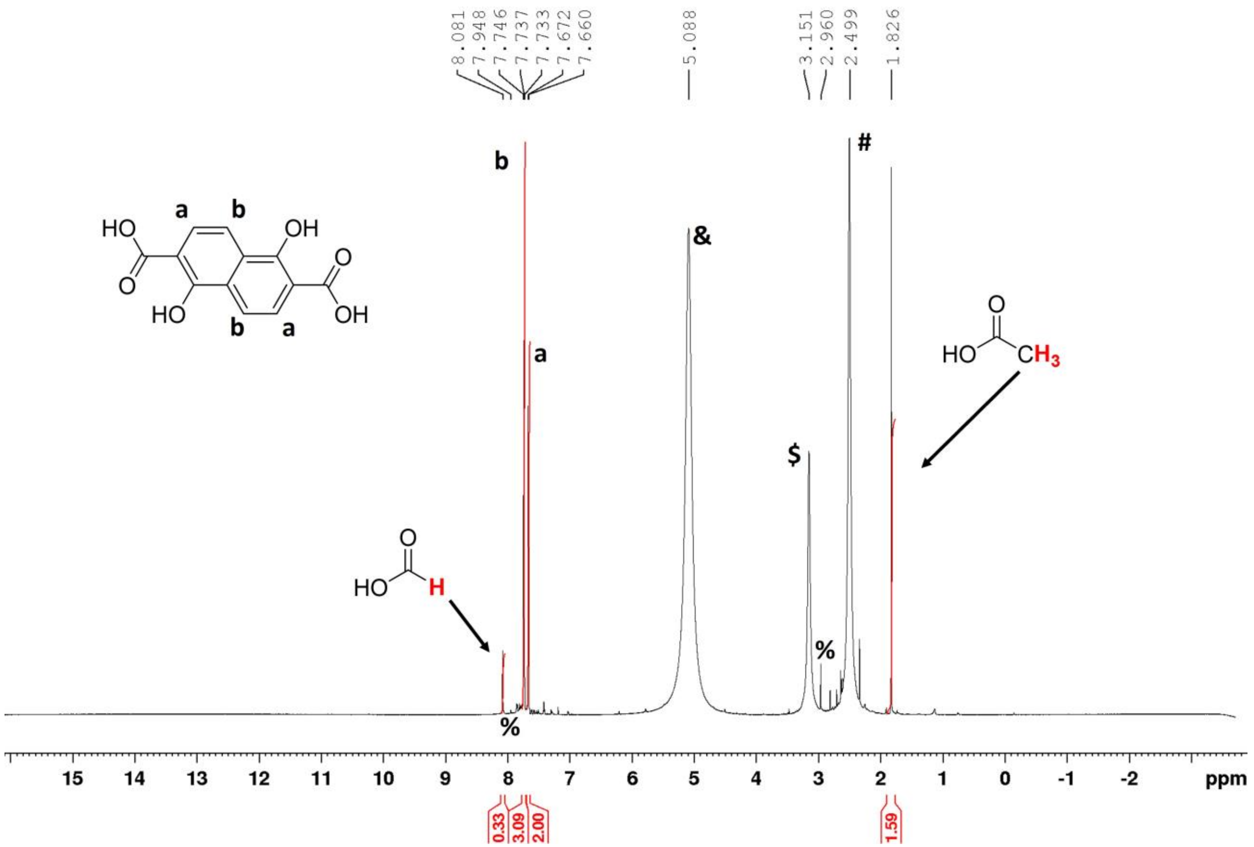

Figure S14. ^{1}H NMR Ni_2dondc diluted in a mixture of dmso-d_6 (marked with #) and DCl (20wt% in D_2O, marked with $). The signal at 5.1 ppm is a superposition of all acidic protons (-OH and -COOH groups, marked with &), stemming from a fast chemical proton exchange. Residual DMF solvent molecules are marked with %. The remaining DMF signal (methyl group) is not visible due to signal overlap. In addition, traces of formic acids are found, stemming from partial thermal decomposition of DMF during the desolvation procedure.

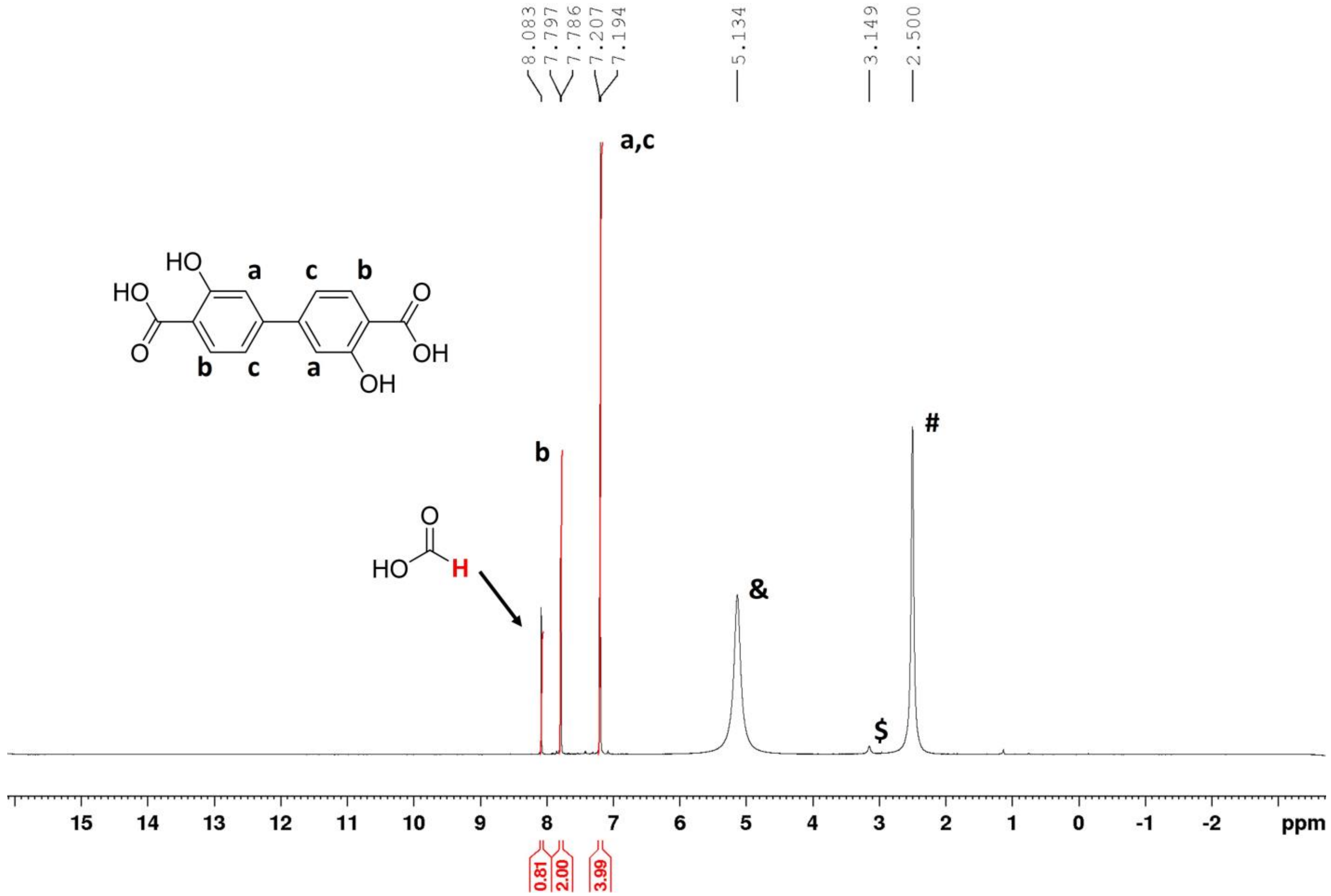

Figure S15. ^{1}H NMR Ni_2bpp diluted in a mixture of dmso-d_6 (marked with #) and DCl (20wt% in D_2O, marked with $). The signal at 5.1 ppm is a superposition of all acidic protons (-OH and -COOH groups, marked with &), stemming from a fast chemical proton exchange.

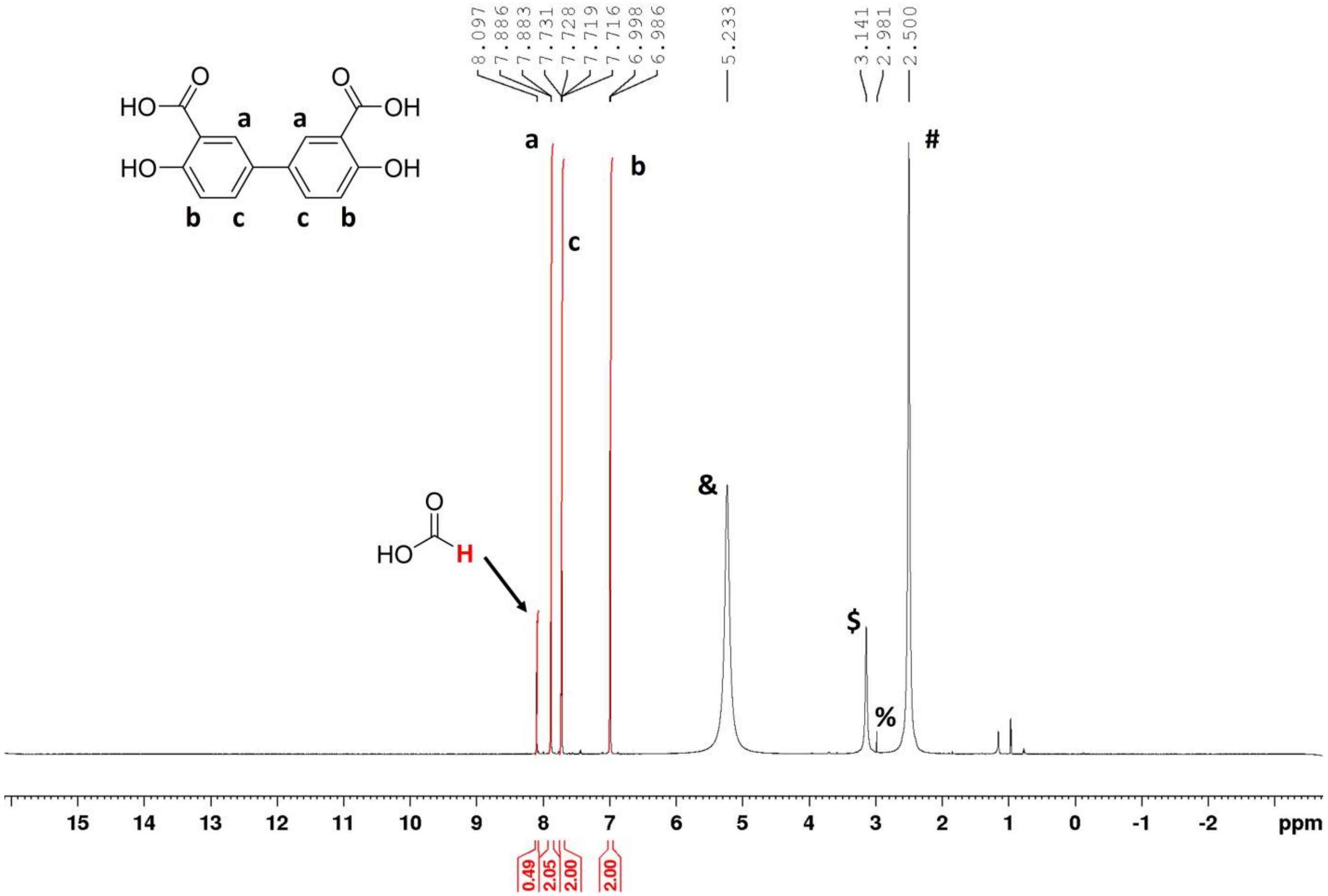

Figure S16. ^{1}H NMR Ni_2bpm diluted in a mixture of dmso-d_6 (marked with #) and DCl (20wt% in D_2O, marked with $). The signal at 5.1 ppm is a superposition of all acidic protons (-OH and -COOH groups, marked with &), stemming from a fast chemical proton exchange. Residual DMF solvent molecules are marked with %. The remaining two DMF signals are not visible due to signal overlap and low relative intensities.

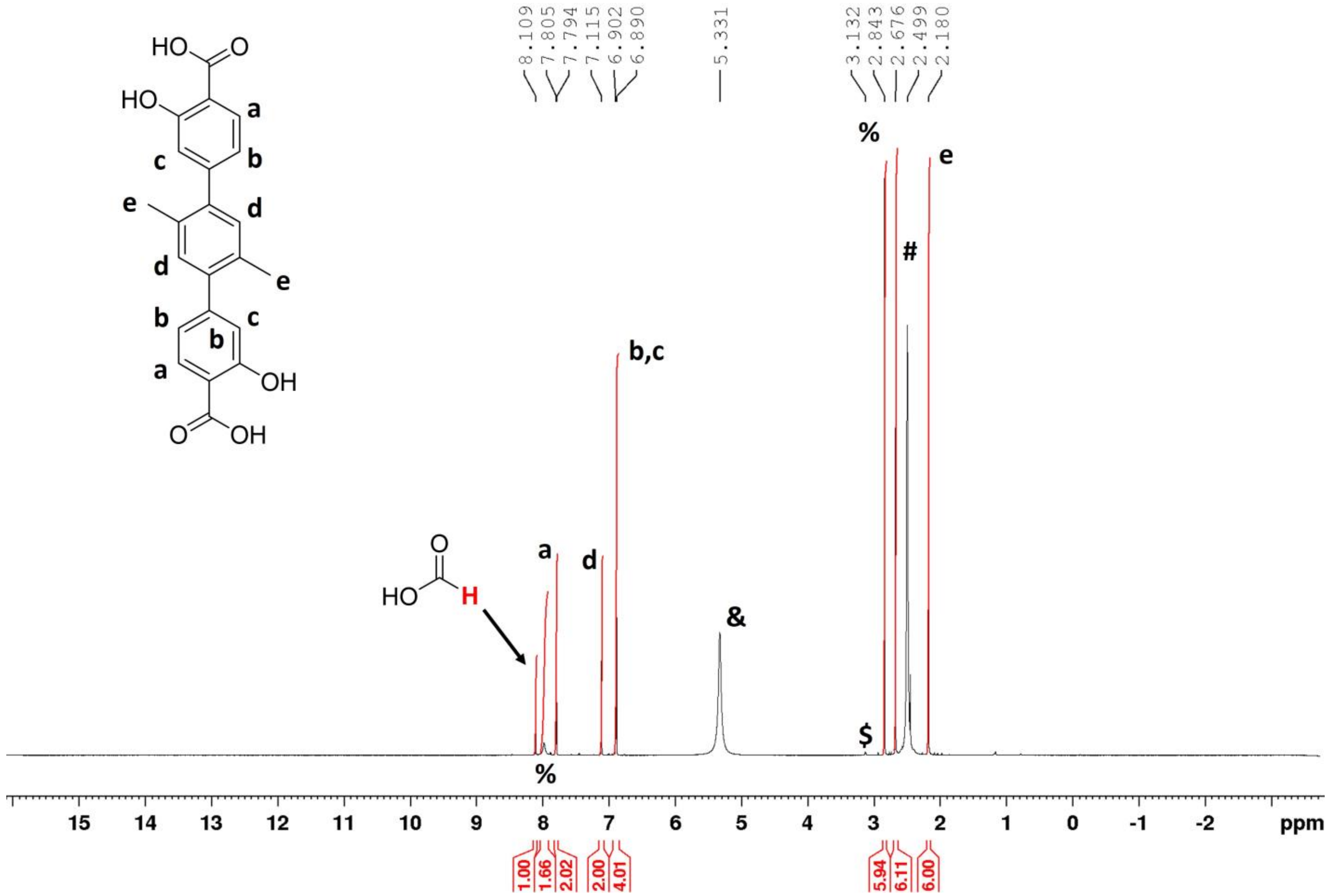

Figure S17. ^{1}H NMR Ni_2tpp diluted in a mixture of dmso-d_6 (marked with #) and DCl (20wt% in D_2O, marked with $). The signal at 5.1 ppm is a superposition of all acidic protons (-OH and -COOH groups, marked with &), stemming from a fast chemical proton exchange. Residual DMF solvent molecules are marked with %.

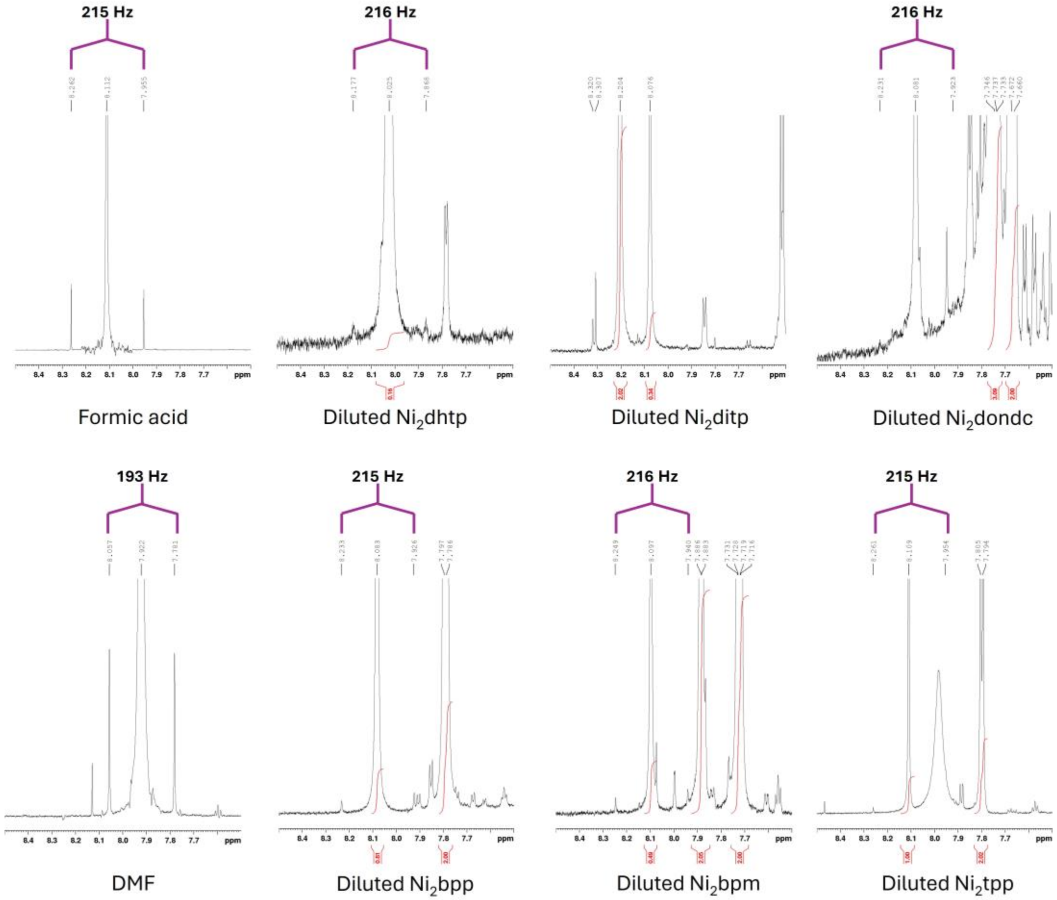

Figure S18. ^{1}H NMR of diluted Ni_2*L* frameworks in the region from 8.5 to 7.5 ppm in comparison with formic acid and DMF, showing the $^{1}J_{CH}$ coupling constants of the ^{13}C satellites, highlighting that the signal at *ca.* 8.1 originates from formate ions. No satellites were found for Ni_2dhip.

TGA (SUPPORTING DATA)

Table S1. Total water uptake determined from the first water mass loss step of the TGA data for hydrated Ni_2L materials.

	$n_{water,TGA}$ (mmol g^{-1})	$n_{water,TGA}$* (mmol mmol^{-1} of MOF)
Ni_2dhtp	20.84 ± 0.14	6.35 ± 0.93
Ni_2dhip	20.95 ± 2.13	6.27 ± 0.20
Ni_2dondc	21.14 ± 2.89	7.91 ± 0.06
Ni_2bpp	21.06 ± 1.48	7.16 ± 0.98
Ni_2bpm	24.97 ± 2.39	8.77 ± 1.13
Ni_2tpp	23.22 ± 1.41	9.30 ± 1.44

* based on sum formulas determined by solution ^{1}H NMR

FTIR (SUPPORTING DATA)

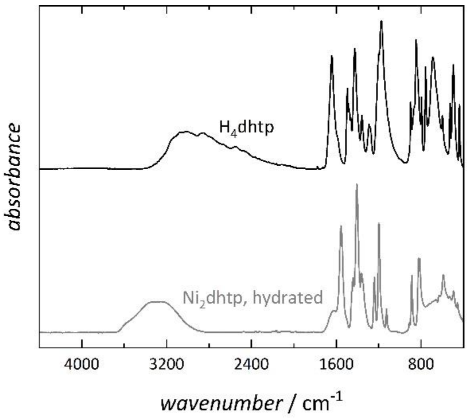

Figure S19. FTIR spectra of hydrated Ni_2dhtp in comparison with spectra of the H_4dhtp linker molecule. The spectra are normalized and vertically offset.

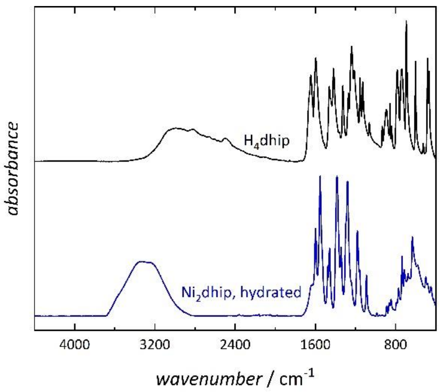

Figure S20. FTIR of hydrated Ni_2dhip in comparison with spectra of the H_4dhip linker molecule. The spectra are normalized and vertically offset.

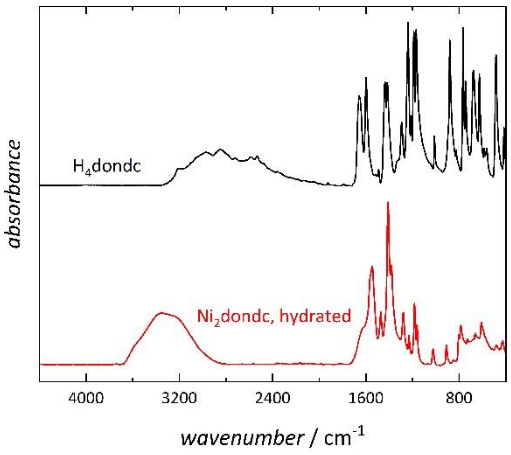

Figure S21. FTIR spectra of hydrated Ni_2dondc in comparison with spectra of the H_4dondc linker molecule. The spectra are normalized and vertically offset.

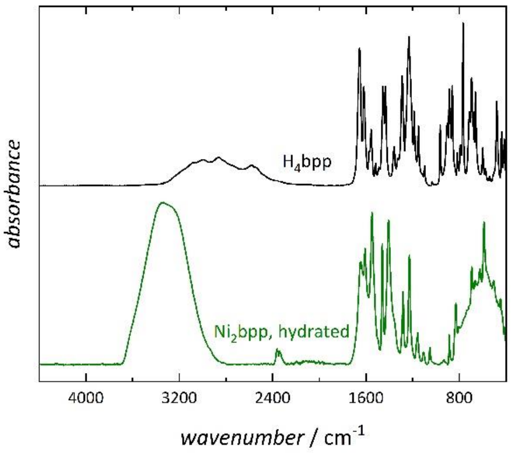

Figure S22. FTIR spectra of hydrated Ni_2bpp in comparison with spectra of the H_4bpp linker molecule. The spectra are normalized and vertically offset.

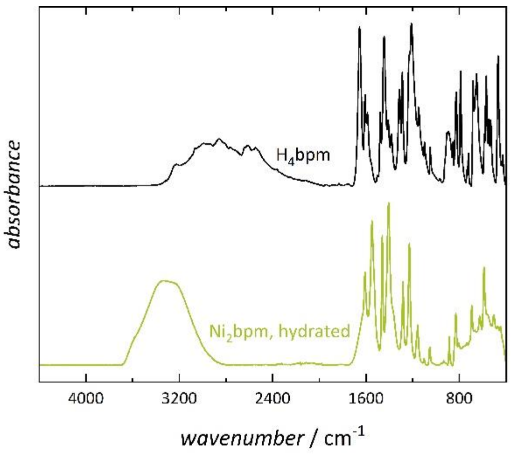

Figure S23. FTIR spectra of hydrated Ni_2bpm in comparison with spectra of the H_4bpm linker molecule. The spectra are normalized and vertically offset.

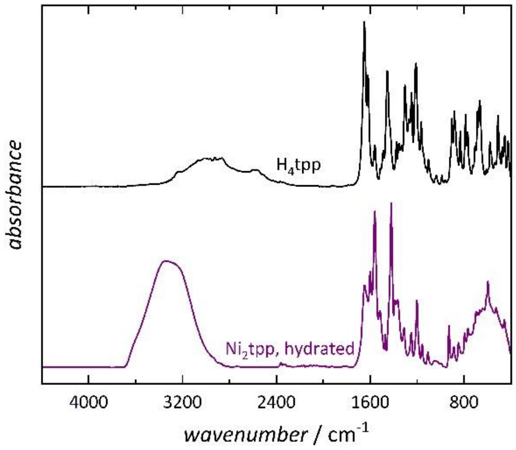

Figure S24. FTIR spectra of hydrated Ni_2tpp in comparison with spectra of the H_4tpp linker molecule. The spectra are normalized and vertically offset.

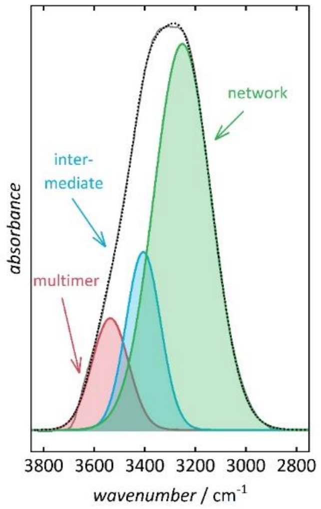

Figure S25. FTIR spectra of pure HPLC grade water, showing the three different vibrational modes of the O-H stretching vibration. The doted lines represent the cumulative fit of the three distinct water bending vibration modes.

Table S2. Results of the least-square fits, showing the contribution of the different vibrational modes to the water stretching vibration for hydrated Ni_2*L* materials (two or three independently synthesized samples).

	**multimer water**		**intermediate water**		**network water**	
	***area* / %**	***error***	***area* / %**	***error***	***area* / %**	***error***
Ni_2dhtp	5.4	0.2	21.6	0.6	73.1	0.4
Ni_2dhip	5.8	0.6	20.3	2.9	73.9	3.0
Ni_2dondc	4.8	0.1	18.1	2.3	77.1	2.3
Ni_2bpp	5.0	0.2	19.6	1.1	75.4	1.0
Ni_2bpm	5.3	1.0	17.7	1.5	77.0	1.8
Ni_2tpp	6.0	0.4	16.5	0.5	77.5	0.1
water	12.8	1.9	18.4	3.2	68.8	1.7

Table S3. Peak center positions and their errors obtained by the least-square fits of the different modes of the water stretching vibration for hydrated Ni_2L materials (two or three independently synthesized samples).

	**multimer water**		**intermediate water**		**network water**	
	***wavenumber* / cm⁻¹**	***error***	***wavenumber* / cm⁻¹**	***error***	***wavenumber* / cm⁻¹**	***error***
Ni_2dhtp	3565.1	0.8	3419.1	0.7	3228.9	2.4
Ni_2dhip	3573.6	0.8	3431.4	0.8	3248.2	1.6
Ni_2dondc	3573.7	0.8	3432.4	0.8	3250.7	1.5
Ni_2bpp	3574.5	1.2	3433.5	0.9	3253.4	1.4
Ni_2bpm	3573.7	1.2	3431.0	0.8	3253.1	1.3
Ni_2tpp	3571.3	1.7	3431.3	1.1	3262.2	1.5
water	3535.9	2.6	3405.8	1.3	3251.6	0.7

WATER SORPTION (SUPPORTING DATA)

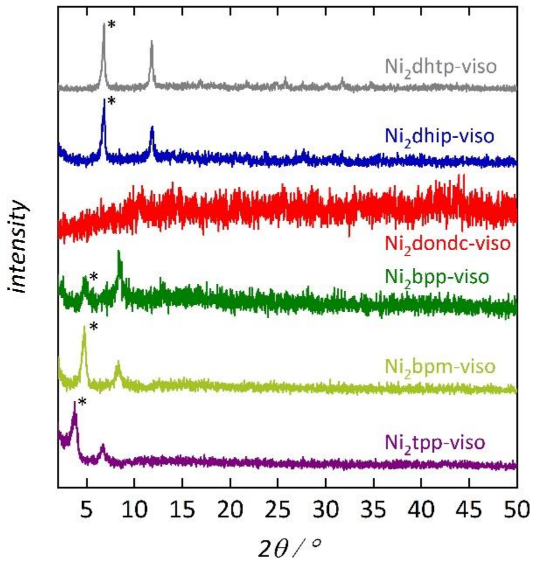

Figure S26. XRD patterns of re-isolated Ni_2L samples, previously used for water vapor sorption measurements. The (110) lattice plane is marked with an asterisk (*).

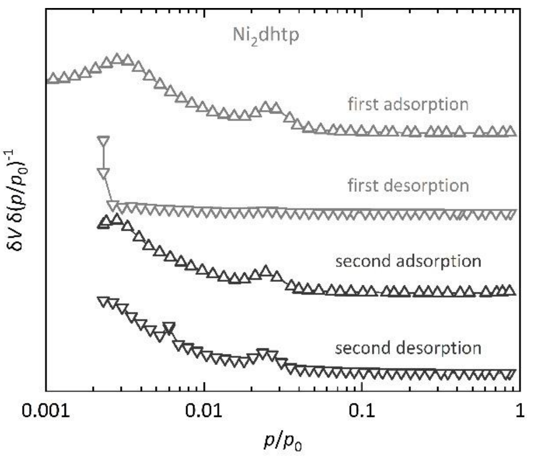

Figure S27. Normalized first derivatives (δV/δ(p/p_0)) of the water vapor sorption
isotherms (25 °C) of Ni_2dhtp.

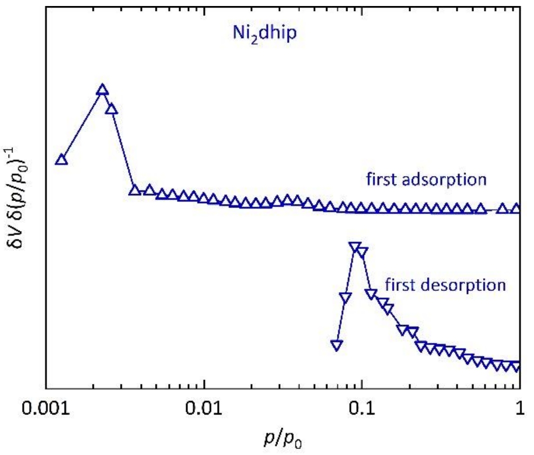

Figure S28. Normalized first derivatives (δV/δ(p/p_0)) of the water vapor sorption
isotherms (25 °C) of Ni_2dhip.

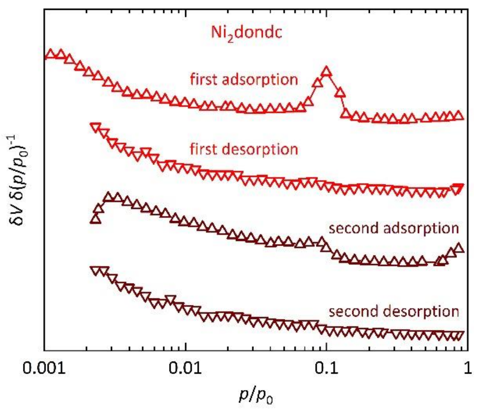

Figure S29. Normalized first derivatives (δV/δ(p/p_0)) of the water vapor sorption
isotherms (25 °C) of Ni_2dondc.

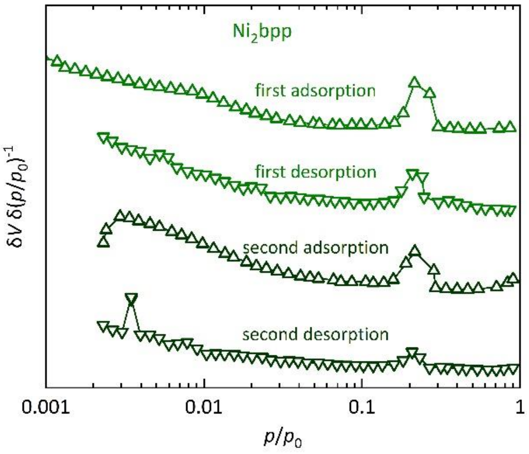

Figure S30. Normalized first derivatives (δV/δ(p/p_0)) of the water vapor sorption
isotherms (25 °C) of Ni_2bpp.

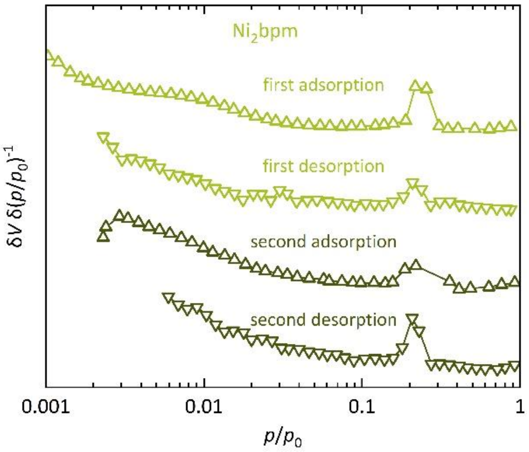

Figure S31. Normalized first derivatives (δV/δ(p/p_0)) of the water vapor sorption
isotherms (25 °C) of Ni_2bpm.

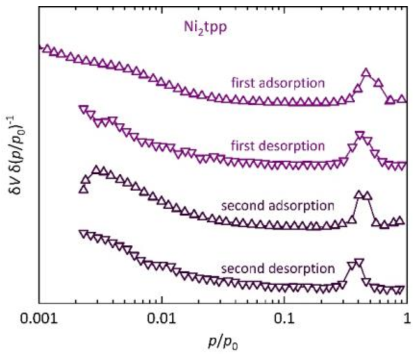

Figure S32. Normalized first derivatives (δV/δ(p/p_0)) of the water vapor sorption
isotherms (25 °C) of Ni_2tpp.

Table S4. Determined total water uptake after the first adsorption branch $n_{water,total}$ determined from water vapor sorption data.

	$n_{water,total}$ **(mmol g⁻¹)**	$n_{water,total}$* **(mmol mmol⁻¹ of MOF)**
Ni_2dhtp	29.89 ± 0.96	9.11 ± 1.34
Ni_2dhip	22.69 ± 3.27	6.92 ± 0.23
Ni_2dondc	24.13 ± 1.43	7.55 ± 0.06
Ni_2bpp	31.67 ± 1.20	10.78 ± 1.48
Ni_2bpm	41.82 ± 4.00	14.46 ± 1.86
Ni_2tpp	47.91 ± 4.04	19.19 ± 2.96

* based on sum formulas determined by solution ^{1}H NMR

Table S5. Octanol-water partition coefficients *log* (P_{OW}) of the H_4L linker molecules, obtained from the ChemDraw (*V21.0.0*) software package.

	log P_{OW}
H_4dhtp	0.37
H_4dhip	0.37
H_4dondc	1.37
H_4bpp	2.04
H_4bpm	2.04
H_4tpp	4.69

Table S6. Comparison of the maximum adsorption of the nitrogen and water sorption isotherms during the first desorption branches.

	$V_{nitrogen}$ **(cm^3 g^{-1})**	V_{water} **(cm^3 g^{-1}) At** p/p_0 **0.55**	V_{water} / $V_{nitrogen}$
Ni_2dhtp	0.55 ± 0.07	0.48 ± 0.01	0.87 ± 0.01
Ni_2dhip	0.38 ± 0.09	0.37 ± 0.06	0.97 ± 0.17
Ni_2dondc	0.55 ± 0.03	0.36 ± 0.01	0.66 ± 0.02
Ni_2bpp	0.74 ± 0.02	0.50 ± 0.03	0.67 ± 0.04
Ni_2bpm	0.58 ± 0.02	0.62 ± 0.04	1.08 ± 0.08
Ni_2tpp	1.03 ± 0.06	0.78 ± 0.04*	0.75 ± 0.04

* pore volume determined at p/p_0 0.7

References

[1] Dietzel, P. D. C.; Georgiev, P. A.; Frøseth, M.; Johnsen, R. E.; Fjellvåg, H.; Blom, R. Effect of Larger Pore Size on the Sorption Properties of Isoreticular Metal–Organic Frameworks with High Number of Open Metal Sites *Chem. – A Eur. J.* **2020**, *26*, 13523–13531. 10.1002/chem.202001825.

[2] Schukraft, G. E. M.; Ayala, S.; Dick, B. L.; Cohen, S. M. Isoreticular expansion of polyMOFs achieves high surface area materials *Chem. Commun.* **2017**, 53, 10684–10687. 10.1039/C7CC04222A

[3] Zheng, J.; Vemuri, R. S.; Estevez, L.; Koech, P. K.; Varga, T.; Camaioni, D. M.; Blake, T. A.; Mcgrail, B. P.; Motkuri, R. K. Pore-Engineered Metal–Organic Frameworks with Excellent Adsorption of Water and Fluorocarbon Refrigerant for Cooling Applications *J. Am. Chem. Soc.* **2017**, *139*, 10601–10604. 10.1021/jacs.7b04872

[4] Heidary, N.; Chartrand, D.; Guiet, A.; Kornienko, N. Rational incorporation of defects within metal–organic frameworks generates highly active electrocatalytic sites *Chem. Sci.* **2021**, *12*, 7324–7333. 10.1039/D1SC00573A.

[5] Kapelewski, M. T.; Geier, S. J.; Hudson, M. R.; Stück, D.; Mason, J. A.; Nelson, J. N.; Xiao, D. J.; Hulvey, Z.; Gilmour, E.; Fitzgerald, S. A.; Head-Gordon, M.; Brown, C. M.; Long, J. R. M_2(*m*-dobdc) (M = Mg, Mn, Fe, Co, Ni) Metal–Organic Frameworks Exhibiting Increased Charge Density and Enhanced H_2 Binding at the Open Metal Sites *J. Am. Chem. Soc.* **2014**, *136*, 12119–12129. 10.1021/ja506230r

[6] Deng, H.; Grunder, S.; Cordova, K. E.; Valente, C.; Furukawa, H.; Hmadeh, M.; Gándara, F.; Whalley, A. C.; Liu, Z.; Asahina, S.; Kazumori, H.; O'Keeffe, M.; Terasaki, O.; Stoddart, J. F.; Yaghi, O. M. Large-Pore Apertures in a Series of Metal-Organic Frameworks *Science* **2012**, *336*, 1018–1023. 10.1126/science.1220131
